# Suppression of Interfacial Loss Pathways at Self-Assembled Molecular Hole Transport Layers in Perovskite Solar Cells

**DOI:** 10.1021/acsami.6c02006

**Published:** 2026-04-21

**Authors:** Shivam Singh, Raquel Dantas Campos, Marielle Deconinck, Elena Siliavka, Vladimir Shilovskikh, Dmitrii Sychev, Ilka M. Hermes, Nir Tessler, Boris Rivkin, Yana Vaynzof

**Affiliations:** † Chair for Emerging Electronic Technologies, TUD Dresden University of Technology, Nöthnitzer Str. 61, Dresden 01187, Germany; ‡ 28394 Leibniz-Institute for Solid State and Materials Research Dresden, Helmholtzstraße 20, Dresden 01069, Germany; § 28408 Leibniz Institut Für Polymerforschung Dresden e.V., Hohe Straße 6, Dresden 01069, Germany; ∥ Sara and Moshe Zisapel Nanoelectronic Center, Electrical and Computer Engineering, 26747 Technion Israel Institute of Technology, Haifa 32000003, Israel

**Keywords:** perovskite solar cells, hole transport layers, buried interface, stability, conductivity

## Abstract

The development of stable and efficient perovskite solar cells (PSCs) hinges on the optimization of interfacial energetics and suppression of parasitic loss pathways. While poly­[bis­(4-phenyl)­(2,4,6-trimethylphenyl)­amine] (PTAA) and [2-(3,6-dimethoxy-9H-carbazol-9-yl)­ethyl] phosphonic acid (MeO-2PACz) are among the most effective hole transport layers (HTLs) for inverted PSC architectures, each presents trade-offs between operational and reverse-bias stability. This study introduces a strategy to form a composite HTL comprising PTAA and MeO-2PACz that synergistically integrates both materials’ advantages while overcoming their limitations. The composite HTL modulates the buried interface to the perovskite, effectively suppressing loss pathways and enhancing the uniformity of the HTL conductivity. Devices incorporating the composite HTL achieve a champion power conversion efficiency (PCE) of 22.83% without additional surface passivation, surpassing the ∼21% achieved by reference devices made using MeO-2PACz or PTAA alone. Moreover, they demonstrate exceptional operational durability and a markedly enhanced reverse bias tolerance. Accompanying drift-diffusion device simulations suggest a previously unexplored loss mechanism at molecular hole transport layers, related to losses induced by electron tunnelling from the perovskite to the hole-collecting contact. Such loss pathways are suppressed when the composite HTL is used, establishing it as a powerful and scalable route toward highly efficient, durable PSCs.

## Graphical abstract

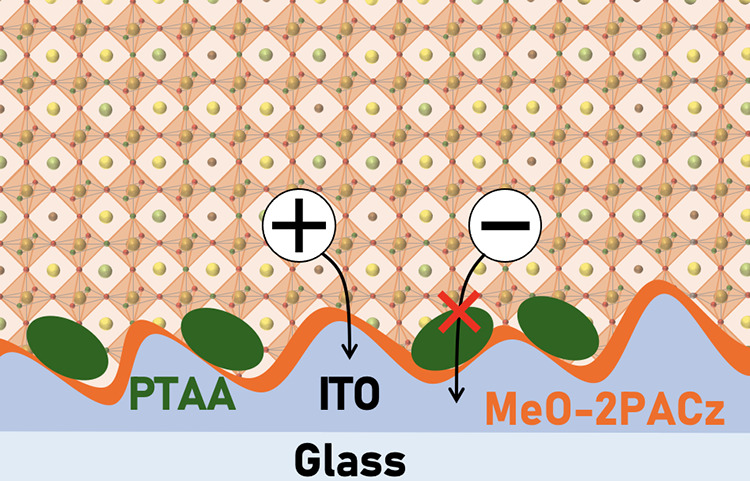

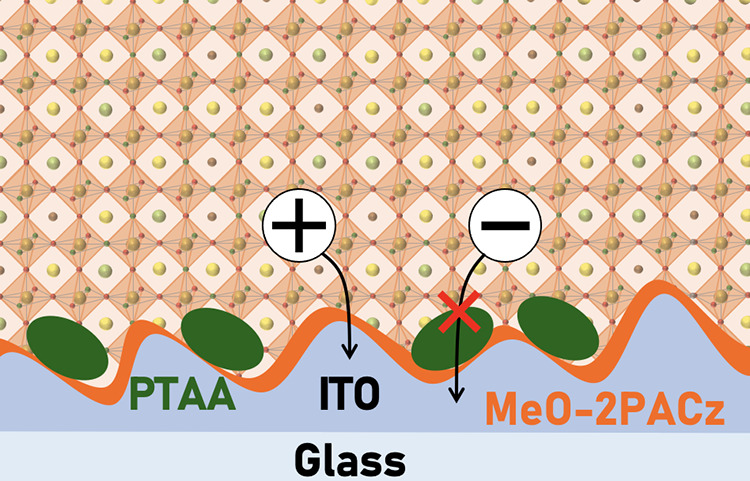

## 1 Introduction

Inverted (p–i–n) architecture perovskite solar cells (PSCs) have emerged as leading candidates for next-generation photovoltaic (PV) technologies, owing to their inherently low hysteresis,^1,2^ compatibility with low-temperature processing,^3–5^ and seamless integration into tandem solar cell designs.^6,7^ A critical factor in realizing the full potential of inverted PSCs is the choice of hole transport layer (HTL), which directly influences charge extraction,^8,9^ interfacial energetics,^10,11^ and long-term device stability.^12,13^ Various HTLs have been explored, including conducting polymers such as poly­(3,4-ethylenedioxythiophene)/poly­(styrenesulfonate) (PEDOT/PSS),^14,15^ transition metal oxides (e.g., NiO_*x*_, VO_*x*_),^16,17^ inorganic compounds (e.g., CuI, CuS, CuSCN),^18,19^ and organic semiconductors like Poly­[*N*,*N*′-bis­(4-butylphenyl)-*N*,*N*′-bisphenylbenzidine] (Poly-TPD)^20^ and poly­[bis­(4-phenyl)­(2,4,6-trimethylphenyl)­amine] (PTAA).^21,22^ In recent years, self-assembled molecular layers (SAMs), particularly those based on carbazole-phosphonic acid derivatives, have gained significant attention for their ability to form well-defined, chemically tailored interfaces.^23,24^ Among the most widely adopted HTLs in high-efficiency inverted PSCs are PTAA and [2-(3,6-dimethoxy-9H-carbazol-9-yl)­ethyl] phosphonic acid (MeO-2PACz), both of which have enabled record power conversion efficiencies (PCE) exceeding 25%.^25,26^ Despite comparable efficiencies, these materials exhibit divergent stability profiles: MeO-2PACz is known for superior operational stability,^27,28^ while PTAA demonstrates enhanced resilience under reverse bias conditions.^29,30^ Given that operational and reverse bias stabilities are crucial for long-term field performance, particularly under maximum power point (MPP) tracking, resolving the trade-offs between these HTLs is paramount for the commercial viability of PSCs.

Ion migration remains a central degradation mechanism under both operational and reverse bias stress, often exacerbated by buried interface defects and inhomogeneities.^31,32^ Our previous studies have demonstrated that the nanoscale texture of the buried HTL/perovskite interface critically influences ion migration pathways and, consequently, device stability.^33^ Since this buried interface is intrinsically linked to the structural and chemical properties of the underlying HTL, engineering this interface offers a promising route for mitigating ion-driven degradation and enhancing PSC longevity. The ultrathin nature of MeO-2PACz layers, typically less than 2 nm, can lead to incomplete surface coverage or microscopic pinholes, particularly on rough indium tin oxide (ITO) substrates.^34,35^ These imperfections may create localized regions where the perovskite absorber can come into direct contact with the bottom electrode, forming undesired loss pathways. Such pathways facilitate nonradiative recombination and current leakage, severely degrading the device’s open-circuit voltage (*V*_OC_), fill factor (FF), and overall PCE. Therefore, mitigating these shunting effects is critical for achieving reliable and high-performance inverted PSCs using MeO-2PACz. An alternative source of performance loss is related to the possibility that the photogenerated electrons from the perovskite layer tunnel through the thin MeO-2PACz layer into the ITO. This may be particularly prominent in those regions where a monolayer of MeO-2PACz is formed. While tunneling of electrons is not often considered when evaluating the interfacial loss mechanisms in perovskite solar cells, it has been recognized as a significant loss mechanism in other types of solar cells.^36^ In contrast, PTAA layers are 7–10 nm thick, thus eliminating both the possibility of forming direct contact and/or electron tunneling to the ITO.^37^ On the other hand, the lack of specific chemical interaction between the PTAA and the perovskite is a disadvantage. As a hydrophobic, nonpolar polymer, PTAA does not form strong interfacial bonds with the ionic components of the perovskite, resulting in a weakly coupled interface. This can lead to poor interfacial contact, inefficient hole extraction, and the formation of interfacial trap states that promote nonradiative recombination.^28^ In contrast, MeO-2PACz contains functional groups, such as phosphonic acid anchors, that form strong covalent bonds with metal oxide surfaces (e.g., ITO) and also promote favorable alignment and contact with the perovskite layer.^27^ This chemical bonding enhances interface passivation and facilitates more efficient charge transfer. These favorable properties, alongside the ability to chemically modify the structure of the SAMs to tailor their energetics, render them particularly promising.^38^

Wang et al. employed a bilayer HTL approach to enhance the performance of micrometer-thick PSCs. In this study, a mixture of [4-(3,6-Dimethyl-9*H*-carbazol-9-yl)­butyl] phosphonic acid (Me-4PACz) and MeO-2PACz was first deposited on ITO, followed by a continuous thin additional layer of PTAA over the SAM mixture to form a bilayer HTL.^39^ The bilayer was reported to lead to higher quasi-Fermi level splitting across the perovskite compared to SAM-only HTL and demonstrated lower recombination rates. Beyond the processing complexity of the bilayer approach, which requires several distinct fabrication steps, the compact coating of the SAM by a PTAA layer might lead to certain disadvantages. For example, it has been shown that the SAMs’ aromatic, amine, or carboxyl groups can form Lewis acid–base interactions with Pb^2+^ in the perovskite absorber, reducing nonradiative recombination and defect density.^40^ Creating a continuous and smooth PTAA layer on top of the SAM can reduce or eliminate direct SAM-perovskite interaction, thus limiting the SAMs’ benefits. Additionally, PTAA, as a hydrophobic polymer, can influence the nucleation and growth of the perovskite, potentially causing unfavorable vertical compositional stratification and reducing the device stability.^33^

In this work, we demonstrate that the complementary functionalities of PTAA and MeO-2PACz can be effectively integrated through the rational design of a composite HTL, which combines both materials and is deposited via a single step. This hybrid architecture leverages the strong interfacial bonding and defect passivation offered by MeO-2PACz, while the inclusion of PTAA aggregates serves to suppress potential loss mechanisms typically associated with ultrathin self-assembled monolayers. Consequently, devices based on the composite HTL reach not only improved photovoltaic performance, but also enhanced operational and reverse bias stabilities.

## 2 Results

### 2.1 Photovoltaic Performance and Reverse Bias Stability

In conventional device fabrication, PTAA is typically dissolved in nonpolar solvents such as chlorobenzene (CB) or toluene, whereas MeO-2PACz requires a polar solvent like ethanol to be dissolved.^27^ However, due to their distinct solubility profiles, PTAA is insoluble in ethanol, and MeO-2PACz is insoluble in CB or toluene, precluding their direct blending in a common solvent system. While MeO-2PACz dissolves readily in *N*,*N*-dimethylformamide (DMF), PTAA exhibits only limited solubility in DMF. Overcoming the PTAA solubility challenge is possible by utilizing a mixed solvent system comprising DMF and CB in a volumetric ratio of 4:1. The PTAA-MeO-2PACz composite HTLs were prepared by mixing the two materials in various volumetric ratios, as detailed in the experimental section.

To evaluate the influence of HTL composition on device performance, inverted PSCs with the architecture ITO/HTL/perovskite/PCBM/BCP/Ag were fabricated (Figure 1a). A series of composite HTLs were tested, denoted as PTAA_*X*_MeO-2PACz_100‑*X*_, where *X* = 100, 75, 50, 25, and 0, corresponding to the volumetric percentage of PTAA in the mixture. All devices were fabricated with triple cation perovskite composition, Cs_0_._05_(MA_1/6_FA_5/6_)_0_._95_Pb­(I_0_._9_Br_0_._1_)_3_ (TrCa), and identical electron transport layers. Importantly, no passivation or any other interfacial modifications were utilized during device fabrication in order to isolate the effects of the composite HTL on the device performance.

**Figure 1:**
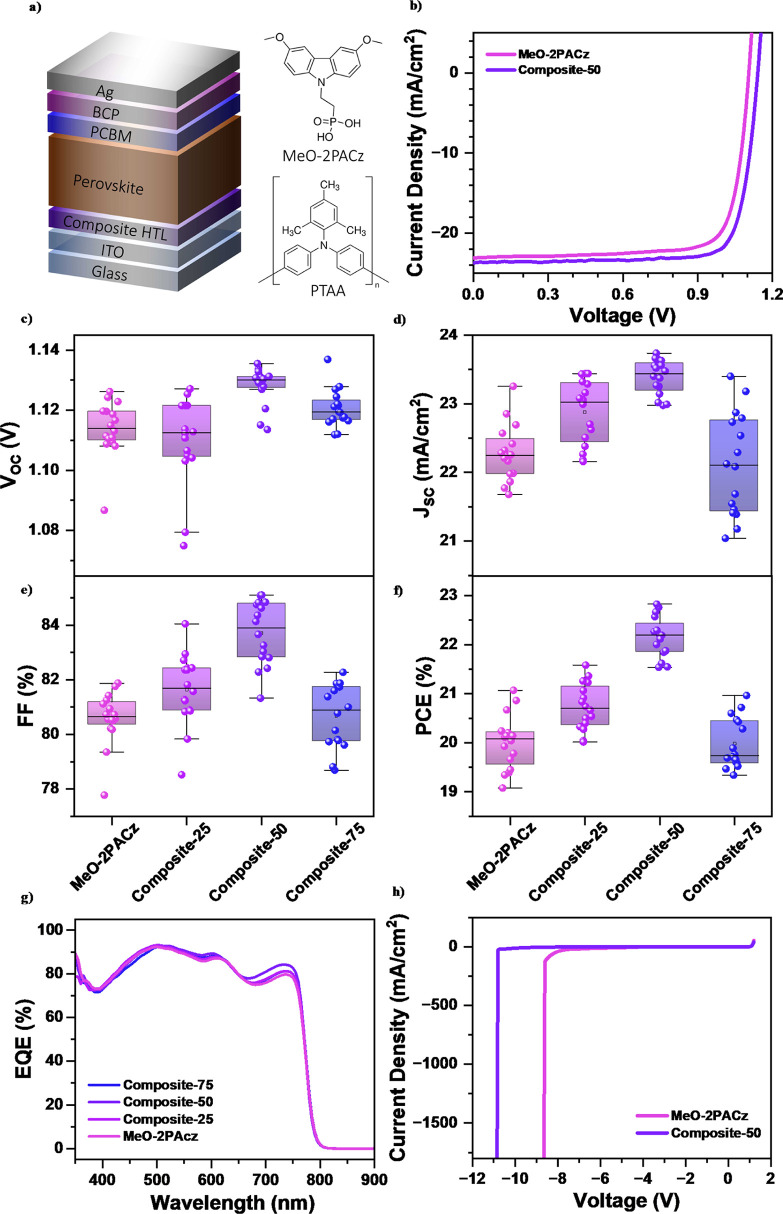
(a) Schematic structure of the inverted architecture-based perovskite solar cell (PSC) with absorber layer Cs_0.05_[(FA)_5/6_(MA)_1/6_]_0.95_Pb­(I_0.9_Br_0.1_)_3_ (TrCa) and chemical structure of PTAA and MeO-2PACz hole transport layers. (b) Illuminated *J*–*V* characteristics of PSC with HTL as MeO-2PACz or composite-50. (c) *V*_OC_, (d) *J*_SC_, (e) FF, (f) PCE, and (g) corresponding EQE of the PSCs with HTL as MeO-2PACz or PTAA-MeO-2PACz composites. (h) Reverse bias stability of PSC with HTL as composite-50 and MeO-2PACz.

Optimization experiments revealed that a MeO-2PACz concentration of 0.75 mg/mL yielded the highest PCE, and this condition was used as the benchmark for all reference cells (Figure S1). Figure 1b presents the current density–voltage (*J*–*V*) characteristics of the champion devices under AM 1.5G illumination (100 mW/cm^2^), highlighting the performance enhancement achieved through HTL compositing. Devices based solely on PTAA demonstrated significantly reduced efficiency (Figure S2), which is attributed to poor HTL film quality stemming from the limited solubility of PTAA in the DMF/CB (4:1) solvent mixture. Figure 1c–f provides a comparative analysis of key photovoltaic parameters such as *V*_OC_, short-circuit current density (*J*_SC_), FF, and PCE. Composite-50 demonstrates superior PV performance relative to other composite compositions and the reference MeO-2PACz. Notably, the enhanced *J*_SC_ observed in PSCs employing the composite-50 HTL is corroborated by an increased external quantum efficiency (EQE) in the 650–750 nm wavelength range (Figure 1g). The champion device based on composite-50 reaches a PCE of 22.83%, significantly surpassing previous reports of triple cation devices in an inverted architecture that do not employ additives and surface passivation (Table S1).

PSCs incorporating MeO-2PACz and composite-50 HTLs were subjected to maximum power point (MPP) tracking to evaluate operational stability. The unencapsulated TrCa PSCs were monitored under continuous MPP tracking for 5 h. Notably, the device with the composite-50 HTL retained approximately 98% of its initial PCE, whereas the MeO-2PACz-based device maintained only 88% over the same duration (Figure S2f). For longer-term stability tests, the devices were encapsulated with UV-curable epoxy resin to prevent moisture-related degradation. MPP tracking over 50 h showed that the composite-50 HTL device retained approximately 100% of its initial PCE, whereas the MeO-2PACz-based device maintained only 89% over the same period (Figure S3). Both the short-term MPP tracking of unencapsulated devices and the longer-term MPP tracking of the encapsulated devices show that the composite-50 HTL exhibits better operational stability than the reference MeO-2PACz HTL.

In recent years, reverse bias stability has emerged as a critical factor alongside operational stability in determining the long-term reliability of PSCs.^40^ Reverse bias stress commonly arises in serially connected modules when low-performing cells (e.g., due to partial shading) are forced to conduct the same current as higher-performing counterparts, potentially leading to irreversible damage.^41^ Enhancing the reverse bias breakdown voltage (*V*_RB_) is a key strategy for improving device robustness under such conditions.^42^ However, inverted PSCs employing MeO-2PACz as the HTL typically exhibit low *V*_RB_ values, often below −1 V.^30^ Figure 1h presents the dark *J*–*V* characteristics of PSCs fabricated with MeO-2PACz and composite-50 as HTLs. The composite-50-based device exhibits a significantly improved *V*_RB_ of −10.8 V, compared to −8.6 V for the MeO-2PACz-based counterpart. The device architecture mirrors that used in Figure 1, except that Au replaces the Ag top electrode. Notably, the *V*_RB_ of the MeO-2PACz-based PSC is considerably higher than previously reported values (e.g., −2.3 V) for similar configurations using Au electrodes.^29,30^ It could be either due to a different perovskite system or the solubility of MeO-2PACz in DMF instead of ethanol or isopropanol.

### 2.2 Investigation of Composite-HTL

To investigate the origin of the enhanced device performance, both the top and buried interfaces of the TrCa perovskite films were examined using scanning electron microscopy (SEM) (Figure S4). The top-view SEM images reveal comparable surface structure and grain size across all samples; however, a notable absence of PbI_2_ flakes is observed on the surface of TrCa perovskite films deposited on MeO-2PACz. The buried interface was exposed using the method described in our previous work.^33^ The perovskite buried interface delaminated from all the composite HTLs displays similar morphology as the one delaminated from MeO-2PACz, although an increasing density and size of voids are evident with higher PTAA content in the composite HTL. These interfacial voids are corroborated by the top-view SEM images of PTAA, composite-50, and MeO-2PACz deposited on ITO substrates (Figure S5). SEM and atomic force microscopy (AFM) analyses further indicate that PTAA does not form a uniform film over ITO, instead aggregating into clusters, whose size depends on the amount of PTAA, likely due to its poor solubility in DMF (Figures S4 and S5). This morphological feature aligns with the suboptimal photovoltaic performance of PTAA-based PSCs (Figure S2a). Interestingly, the aggregate size is significantly reduced in the composite-50 HTL, as confirmed by AFM (Figure S5). All TrCa perovskite layers exhibit a uniform thickness of ∼450 nm (Figure S6) and consistent perovskite phase formation (Figure S7) without significant changes in crystallinity or orientation (Figure S8). The X-ray photoemission spectroscopy (XPS) core-level spectra of composite-50 indicate the incorporation of MeO-2PACz; however, no definite evidence of PTAA is observed (Figures S9 and S10). These observations suggest that the composite HTLs do not form a well-defined bilayer; instead, PTAA aggregates are dispersed on top of a continuous MeO-2PACz layer.

To investigate the functional role of PTAA aggregates on MeO-2PACz, a control experiment was conducted in which CB was statically spin-coated onto the composite-50 HTL to selectively remove loosely bound PTAA aggregates prior to perovskite deposition. The PV performance of devices fabricated on this CB-washed composite-50 was then compared with composite-50 and MeO-2PACz-based devices (Figure S11). Notably, the PCE of the CB-washed composite-50 devices decreased and approached the performance level of MeO-2PACz alone, indirectly confirming the beneficial role of PTAA aggregates in enhancing device efficiency.

In addition to morphological effects, interfacial energetics were examined as a contributing factor to the improved PCE. Ultraviolet photoemission spectroscopy (UPS) was performed on composite-50 HTL and MeO-2PACz and TrCa perovskite films deposited on top of these HTLs (Figure S12). Consistent with the XPS findings, UPS measurements revealed that the energetics of the composite-50 HTL were very similar to those of MeO-2PACz, confirming that MeO-2PACz predominantly governs the HTL surface coverage. This further suggests that aggregates do not significantly affect the interfacial energetics. This finding was corroborated by Kelvin probe force microscopy (KPFM),^43^ which revealed nearly identical average surface potentials (Δ*V*_CPD_ = 20 mV) for both HTLs, translating to similar work functions. Despite the comparable average surface potentials observed for composite-50 and MeO-2PACz HTLs, KPFM imaging reveals an important morphological distinction: the composite-50 HTL exhibits a distribution of small PTAA aggregates that are not readily discernible in AFM topography images (Figure 2). InLens SEM imaging (Figure S5) further supports this nanoscale heterogeneity in the surface landscape, highlighting the coexistence of small aggregates alongside larger PTAA clusters. These observations underscore the complex surface architecture of the composite-50 HTL, which may play a pivotal role in modulating interfacial contact and charge extraction, contributing to the enhanced device performance.

**Figure 2:**
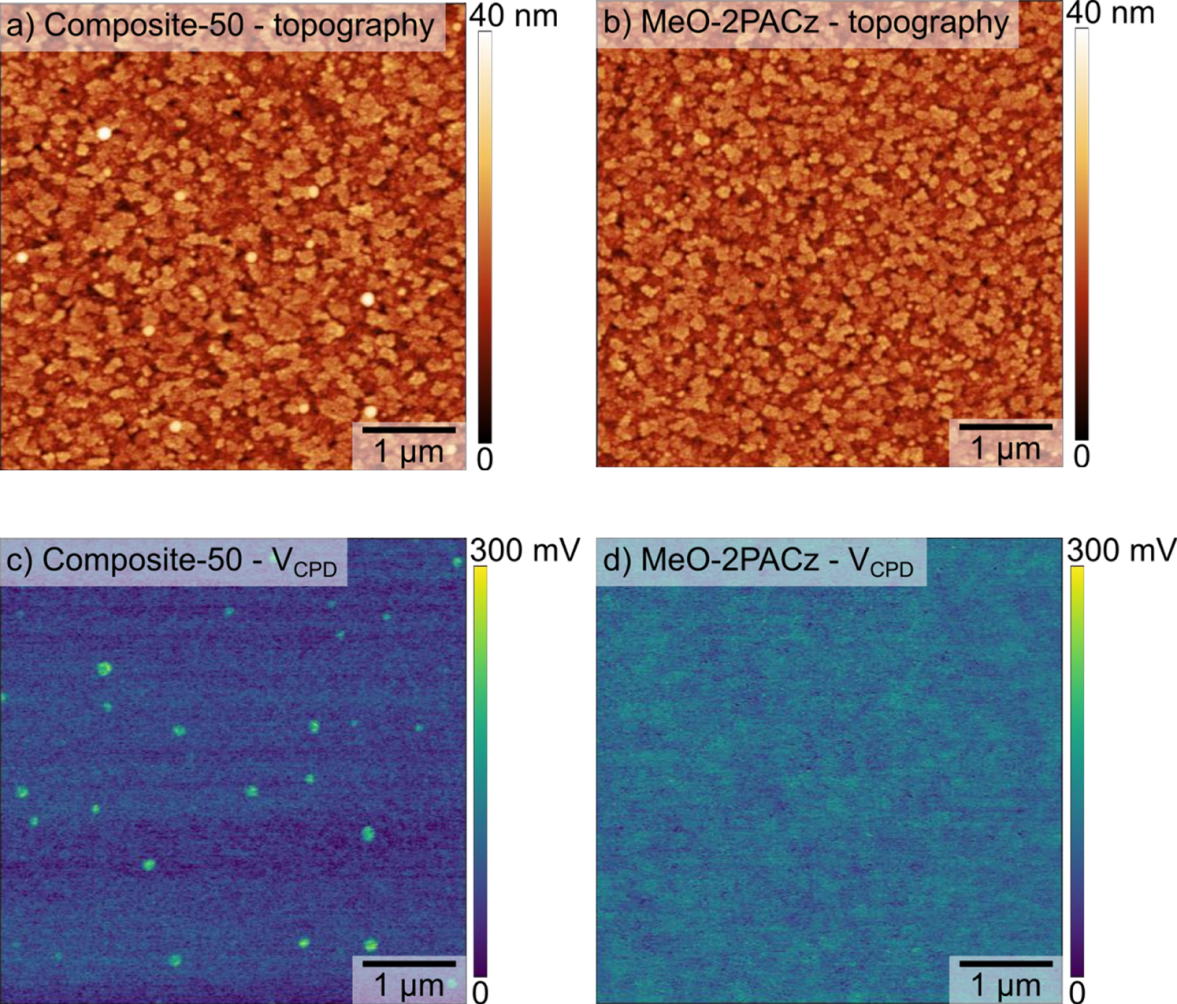
(a,b) AFM topography and (c,d) KPFM surface potential (*V*_CPD_) of the (a,c) composite-50 and (b,d) MeO-2PACz HTLs.

Additionally, conductive-atomic force microscopy (C-AFM) was employed to assess the spatial conductivity of MeO-2PACz and composite-50 HTLs (Figure 3).^44^ The measurements revealed that the composite-50 HTL exhibited a median current of 0.47 nA across a 25 μm^2^ area, substantially lower than MeO-2PACz (1.12 nA). Moreover, the two HTLs exhibited a significant difference in the width of the current distribution across the imaged area (Figure 3e,f), with MeO-2PACz’s distribution spanning a broader range with a notable contribution of highly conductive pathways. For example, while only ∼4% of the composite-50 sample showed currents surpassing 2 nA, this fraction is ∼18% in the MeO-2PACz-only HTL, most likely a consequence of coverage inhomogeneities of the MeO-2PACz HTL that may form either monolayers or multilayers across the sample. The composite HTL, on the other hand, leads to a far more uniform conductivity across the examined area.

**Figure 3:**
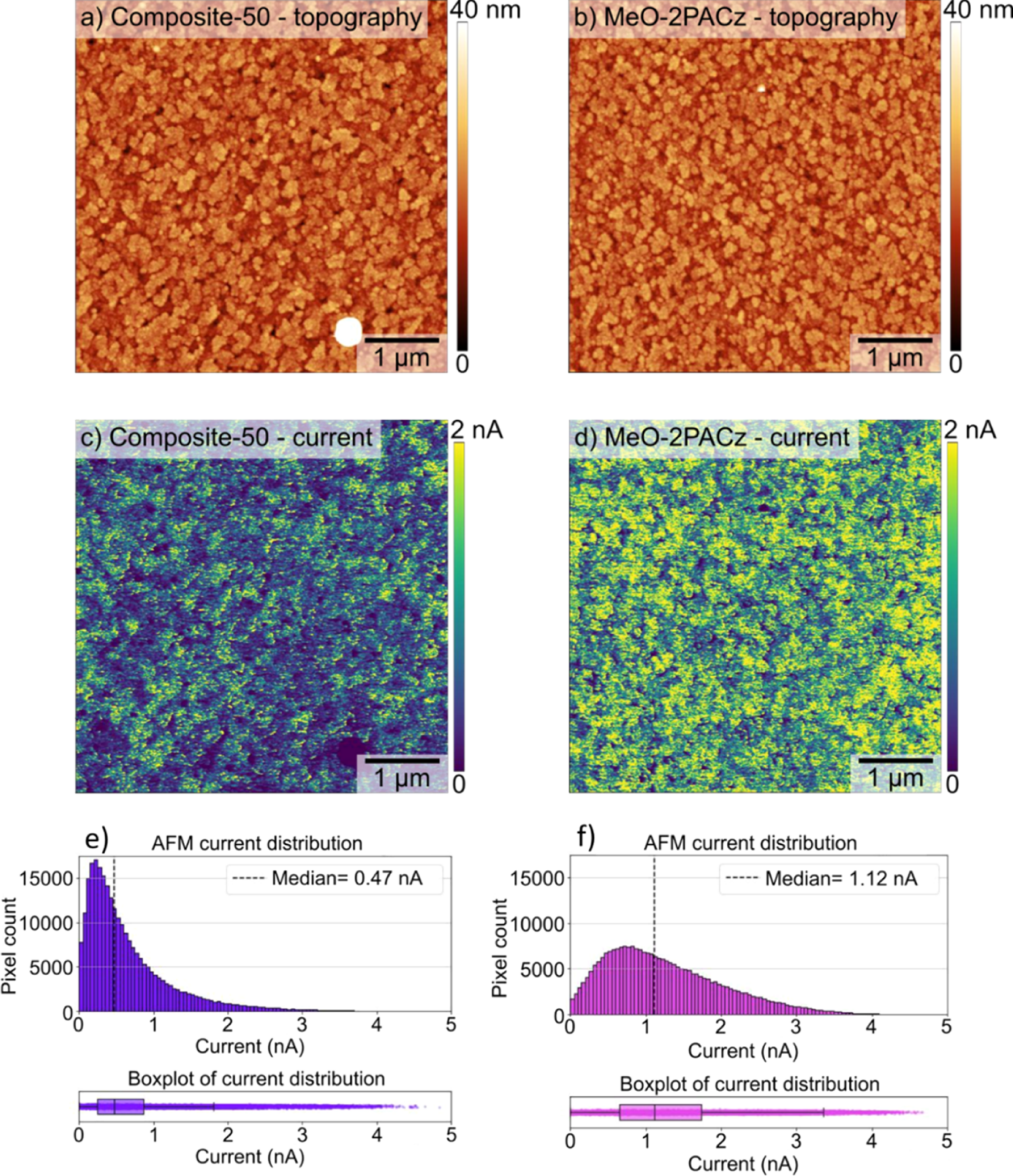
(a,b) AFM topography and (c,d) C-AFM current distribution of the (a,c) composite-50 and (b,d) MeO-2PACz HTLs. The current distribution histogram for (e) composite-50 and (f) MeO-2PACz HTLs.

Steady-state photoluminescence (PL) spectroscopy was performed on TrCa perovskite films using a 405 nm diode laser, with excitation delivered either from the film side or through the ITO/glass substrate. Among the three hole-transport layers (HTLs) examined, the film deposited on ITO with few aggregates of PTAA exhibited the highest PL intensity, followed by MeO-2PACz and the composite-50 layer (Figure S13b). This trend became even more pronounced under backside (ITO/glass) excitation (Figure S13c). The strong PL emitted by the PTAA sample reflects the discontinuous, aggregate nature of the PTAA film, which fails to provide an efficient pathway for hole extraction. Consequently, a larger population of photogenerated holes remains in the perovskite bulk and recombines radiatively with electrons. In contrast, the MeO-2PACz and composite-50 interlayers facilitate more effective hole extraction, leaving fewer carriers available for radiative recombination and thus suppressing the PL signal.^45^ Therefore, the relatively weakest PL from the TrCa/composite-50 stack signifies the most efficient hole extraction of the three HTLs. To further validate the efficient hole extraction by the Composite-50 HTL, time-resolved photoluminescence (TRPL) measurements were conducted on Composite-50/TrCa and MeO-2PACz/TrCa films fabricated on glass substrates. The analysis shows that MeO-2PACz/TrCa requires a triexponential fit, with a small long-lived component (∼756 ns) indicative of deep trap states, whereas composite-50/TrCa is well-fitted by a biexponential decay with a dominant fast component (τ_1_ = 124 ns, amplitude fraction = 92%), suggesting efficient hole transfer (Figure S13d and Table S2). In addition, quasi-Fermi level splitting (QFLS) measurements were performed on TrCa perovskite films fabricated on MeO-2PACz and composite-50 HTLs. The QFLS for the composite-50 HTL is 1.156 eV, which is slightly higher than that of MeO-2PACz (1.142 eV) (Figure S14). Taken together, the TRPL, QFLS, and steady-state PL results consistently show more efficient interfacial charge extraction in the Composite-50 HTL, demonstrated by faster carrier dynamics, improved QFLS, and reduced radiative recombination.

To investigate whether PTAA aggregates influence the crystallization dynamics of the perovskite layer, FA_X_MA_1‑X_PbI_3_ devices were fabricated using a two-step sequential deposition method.^46^ In this approach, a PbI_2_ layer was first deposited onto the HTL, followed by the introduction of organic cations to complete perovskite formation. Detailed information on the film formation process is provided in the Supporting Information. In contrast to the TrCa perovskite system, where crystallization occurs from both the top (antisolvent treatment) and bottom interfaces (HTL texture),^33^ the two-step sequential deposition method limits cation diffusion from the top surface. This configuration allows for isolating the effect of the HTL on perovskite growth. The composite-50 HTL was benchmarked against two reference layers: MeO-2PACz (processed in DMF) and PTAA (processed in chlorobenzene). Notably, even in this top-diffusion-only system, devices incorporating the composite-50 HTL exhibited superior performance compared to those based on either MeO-2PACz or PTAA (Figure S15). These findings confirm that the PTAA aggregates in the composite-50 do not significantly alter perovskite growth kinetics and represent a diversely applicable strategy for various perovskite compositions.

### 2.3 Drift-Diffusion Simulation

Efficiency loss caused by a nonuniform thin HTL can only be attributed to electron flow to the ITO (i.e., contact recombination), particularly in regions of incomplete coverage or where the HTL thickness allows tunneling. To distinguish between these two scenarios, we will consider the measured fraction of highly conducting regions, which is 4% for Composite-50 and 18% for MeO-2PACz. To investigate whether electron tunneling or direct contact with ITO plays a more dominant role in MeO-2PACz-based devices, we performed drift-diffusion simulations (See Supplementary Note 1 for details) in which the effect of the two possible mechanisms was examined.^47–49^ We highlight that these simulations are not meant to reproduce experimental data, but rather aid in distinguishing between the two possible mechanisms. Figure 4a shows the simulated *J*–*V* curves of devices with different HTL thicknesses for which electron tunneling from the perovskite layer to the ITO contact is enabled. It can be seen that thinner HTL layers lead to a reduction in all photovoltaic parameters, suggesting that electron tunneling is an important loss mechanism for HTL thicknesses below 2.5 nm. We note, however, that the effect is gradual and that a significant difference in tunneling probability (e.g., between 4 and 2.25 nm) does not significantly deteriorate the device performance.

**Figure 4:**
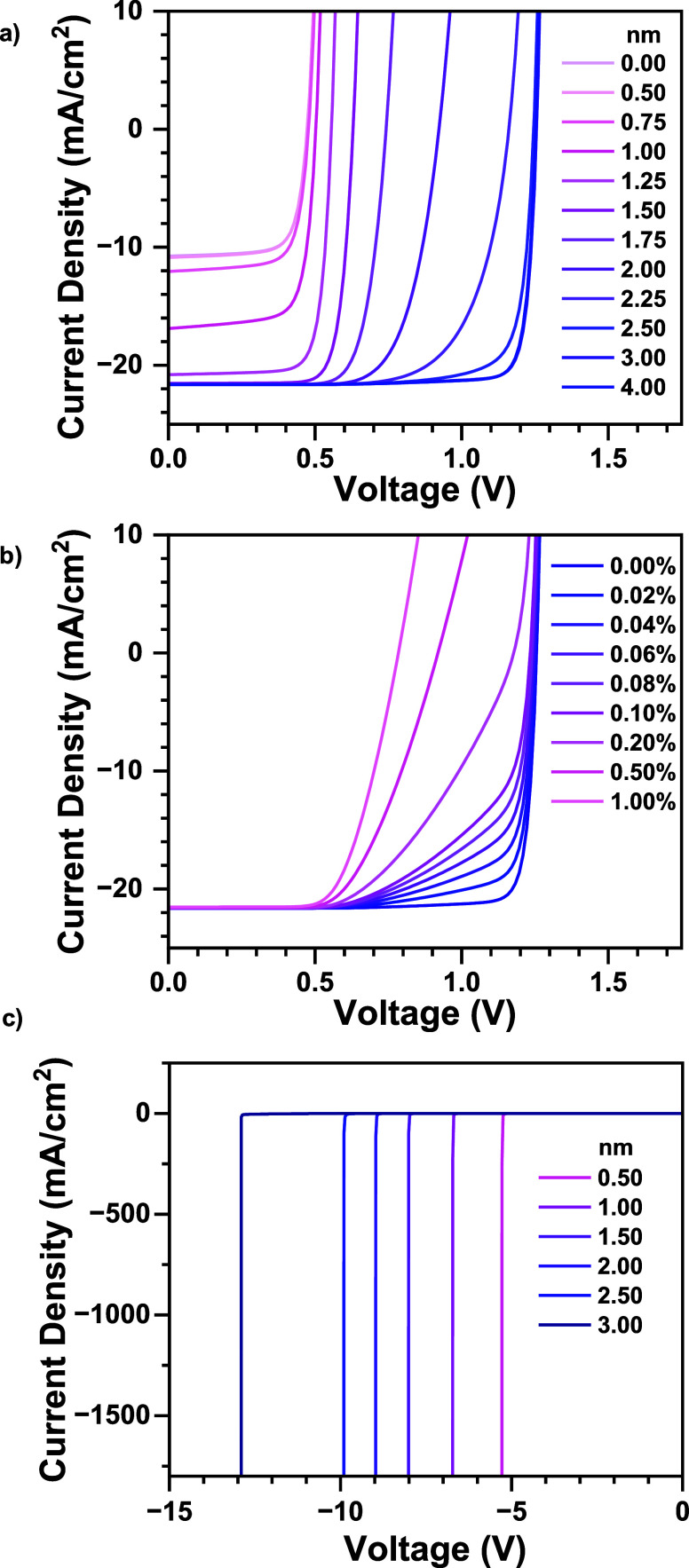
Simulated *J*–*V* of (a) different thicknesses of HTLs with electron tunneling and (b) different fractions of direct contact with ITO. (c) Reverse bias breakdown simulation for different thicknesses of HTLs.

Figure 4b simulates the *J*–*V* curves of devices in which small fractions of the device area are in direct contact with the ITO. Unlike the case of tunneling, it can be seen that such a scenario has a particularly detrimental effect on the FF, which even as little as 0.02% of the active layer area being in contact with ITO leads to a substantial decrease in the FF. Based on the results of these simulations and the relatively high FF of our MeO-2PACz-based devices, we postulate that it is electron tunneling through the thinnest areas of MeO-2PACz that slightly reduces the device performance. The composite HTL leads to a more homogeneous conductivity distribution, in which the highly conductive pathways (thinnest areas of MeO-2PACz) are suppressed. This leads to a reduction in the losses introduced via the tunneling of electrons into the ITO layer. The suppression of the thinnest areas of MeO-2PACs is also consistent with our experimental estimate of the average thickness of the HTLs by performing XPS measurements on both the HTLs and the bare ITO substrate. Using the reduction in In 3d counts in XPS provides an approximate value of HTL thickness, using following equation $\frac{A}{A_{0}}=e^{-d/\lambda }$ , where *A* and *A*_0_ are the integrated area under the In 3d peak for HTLs and ITO, respectively. D is the thickness of the HTLs, and λ is the inelastic mean free path (∼2 nm). The results show that the thickness of the MeO-2PACz HTL is approximately 2.3 nm, while that of the composite-50 is ∼2.7 nm. This slight increase in the average thickness is consistent with our interpretation.

For the reverse breakdown simulations, we added the module of self-heating, where the *I*–*V* joule heating causes a temperature rise. The simulation predicts that the breakdown is due to thermal runaway, with the local temperature rising by 100–200 °C. The results for varying HTL thickness are shown in Figure 4c.

## 3 Discussion

The experimental results clearly demonstrate the improved efficiency of PSCs incorporating the composite HTL (Figure 1f) compared with devices employing the widely used molecular HTL, MeO-2PACz. Previous studies have reported the use of bilayer HTLs (SAM/PTAA) to enhance device performance; however, such approaches typically require complex, multistep fabrication processes.^39^ In contrast, the present work introduces a facile, cost-effective strategy for fabricating a composite HTL that integrates the advantages of both PTAA and MeO-2PACz without necessitating sequential deposition steps.

Comprehensive characterization including AFM (Figure S5), SEM (Figure S6), KPFM (Figure 2), C-AFM (Figure 3), XPS (Figures S9 and S10), and UPS (Figure S12) confirms that the composite layer does not form a conventional bilayer structure. Instead, PTAA aggregates are distributed over a continuous MeO-2PACz layer. Although the ultrathin MeO-2PACz layer benefits from interfacial passivation and energy level alignment, it is vulnerable to electron tunneling losses at the ITO interface. To our knowledge, no existing reports in the literature address this issue. Our approach tackles this problem by adding PTAA aggregates, which help reduce these loss pathways and ensure uniform conductivity across the composite-50 HTL. This architecture significantly reduces interfacial recombination losses and enhances charge transport, thereby improving both device efficiency and operational stability without requiring additional passivation treatments. Although further optimization through additives or surface functionalization could yield incremental gains, this study primarily focuses on identifying and mitigating loss mechanisms intrinsic to ultrathin MeO-2PACz layers via composite HTL engineering.

Drift-diffusion simulations further substantiate the experimental findings, revealing that electron tunneling constitutes a major loss channel for HTL thicknesses below 2.5 nm (Figure 4). The composite HTL achieves a more homogeneous conductivity distribution (Figure 3) by suppressing the highly conductive regions associated with the thinnest areas of MeO-2PACz. This suppression directly contributes to improved operational (Figure S3) and reverse-bias stability (Figures 1h and 4c). Prolonged operation may further reduce the efficiency of both devices, but we believe this will not alter the conclusion that the composite-50 has a stability advantage over MeO-2PACz.

Importantly, we note that the optimization of the HTL/perovskite buried interface is highly complex. For example, while increasing the HTL thickness can help suppress electron tunneling losses, it might not necessarily lead to increased performance. In particular, thick PTAA or NiO_*x*_ layers often lead to lower performance than that achieved using MeO-2PACz. This might be related to a multitude of factors such as the conductivity of the HTL layers, the presence of surface traps (especially for NiO_*x*_), the texture of the HTL and the energetic alignment between the HTL and the perovskite active layer.^10,27,33^ The concurrence of these factors makes it impossible to isolate electron tunneling on its own and to probe it experimentally without the effects of the other factors. On the other hand, the results also consistently show that devices with composite HTLs outperform those made with MeO-2PACz alone. This suggests that the use of MeO-2PACz on its own is, while holding many advantages, not fully optimal. It is important to consider that the MeO-2PACz does not form a perfect self-assembled monolayer, but is rather a combination of monolayers and multilayers formed across the rough ITO substrate. This inheritably suggests that some areas would be more prone to electron tunneling losses. Our drift-diffusion simulations demonstrate that such losses may lead to efficiency loss, yet are not nearly as detrimental as direct contact to ITO. Consequently, the simulations suggest that the suppression of electron tunneling is the most plausible explanation for the observed improvements in device performance and stability for the composite HTLs.

## 4 Conclusion

In summary, we demonstrated a composite HTL by blending PTAA and MeO-2PACz utilizing their complementary properties. The ultrathin nature of the MeO-2PACz layer can result in losses due to electron tunneling to the ITO layer. Incorporating PTAA aggregates within the composite HTL effectively addresses loss pathways, while simultaneously leveraging the interfacial passivation and energy level alignment benefits of MeO-2PACz. As a result, the inverted PSCs employing this composite HTL exhibit enhanced PCE and significantly improved operational and reverse bias stability.

## 5 Experimental Section

### 5.1 Materials

Glass substrates (12 by 12 mm), precoated with a central ITO stripe of 8 by 12 mm, were purchased from PsiOTec Ltd. Formamidinium iodide (FAI, HC­(NH_2_)_2_), and methylammonium iodide (MAI, CH_3_NH_3_I) were purchased from Great Cell Solar Materials. Cesium iodide (CsI), lead iodide (PbI_2_), lead bromide (PbBr_2_) and MeO-2PACz were purchased from TCI. Phenyl-C60-butyricacid methyl ester (PCBM) was purchased from Luminescence Technology Corporation (Lumtec). PTAA and bathocuproine (BCP; sublimed grade,99.99% purity) were purchased from Sigma-Aldrich. All the anhydrous solvents were purchased from Acros Organics. Silver and gold pallets for thermal evaporation of the top contact were purchased from Kurt J. Lesker Company.

### 5.2 HTL Solution Preparation

1.5 mg MeO-2PACz was dissolved in anhydrous DMF and kept overnight stirring at room temperature. The stock MeO-2PACz solution was diluted with DMF for different MeO-2PACz concentration solutions. 1.5 mg PTAA was dissolved in a solvent mixture of DMF/chlorobenzene (4:1) and kept overnight stirring at 100 °C. The prepared solution was filtered through a 0.22 μm polytetrafluoroethylene (PTFE) filter before use. For composite HTLs, the stock solution of MeO-2PACz (1.5 mg/mL) and PTAA (1.5 mg/mL) were mixed in different volumetric ratios and kept at 100 °C for use.

### 5.3 Triple Cation (TrCa) Perovskite Solution Preparation

The perovskite precursor solution (1.2 M) contained mixed cations (Pb, Cs, FA and MA) and halides (I and Br) dissolved in a solvent mixture of DMF/DMSO (4:1) according to the formula of Cs_0.05_[(FA)_5/6_ (MA)_1/6_]_0.95_Pb­(I_0.9_Br_0.1_)_3_ with an excess of 1% PbI_2_.^4,33^

### 5.4 FA_*x*_MA_1–*x*_PbI_3_ Perovskite Solution Preparation

The PbI_2_ solution was prepared by dissolving 816 mg PbI_2_ in 1 mL anhydrous DMF/DMSO (9:1, v/v) solvent mixture. The mixed organic cation solution was prepared by mixing FAI, MAI, and MACl in the amounts of 90, 6.39, and 9 mg, respectively, in 950 μL anhydrous isopropanol.^46^

### 5.5 ETL Solution Preparation

PCBM was dissolved in anhydrous chlorobenzene with a concentration of 20 mg/mL and kept overnight stirring at 70 °C. The prepared PCBM solution was filtered through a 0.22 μm polytetrafluoroethylene (PTFE) filter before use. BCP was dissolved in isopropanol with a concentration of 0.5 mg/mL and kept overnight stirring at 70 °C.

### 5.6 Device Fabrication

The devices were fabricated in an inverted (p–i–n) architecture. ITO coated glass substrates were cleaned with deionized water, acetone, and isopropanol by ultrasonication for 10 min in each solvent. The substrates were then dried with N_2_ and treated with oxygen plasma at 100 mW for 10 min. The substrates were immediately transferred to drybox (relative humidity <1%) for HTL and perovskite deposition. The MeO-2PACz solution was spin-coated at a speed of 3000 rpm for 30 s (with an acceleration of 1500 rpm/s). The hot PTAA solution and composite HTL solutions were spin-coated at a speed of 3000 rpm for 30 s (with an acceleration of 1500 rpm/s). The spin-coated substrates were immediately transferred to a preheated hot plate for annealing at 100 °C for 10 min. The HTL-coated substrates were cooled down on the workbench for 5 min before the deposition of perovskite precursor.

The TrCa perovskite layer was deposited via a two-step spin-coating procedure with 1000 rpm for 10 s and 6000 rpm for 30 s. Anhydrous chlorobenzene (150 μL) was dripped on the spinning substrate during the last 5 s of the second spin-coating step. Subsequently, the spin-coated samples were annealed at 100 °C for 30 min.

For FA_*x*_MA_1–*x*_PbI_3_ perovskite layer, the PbI_2_ solution was spin-coated on ITO/HTL at 2800 rpm for 30 s in a dry air-filled glovebox (relative humidity <1.0%). Then, the PbI_2_ film was transferred into a vacuum chamber for 5 min to remove extra solvent. After the evacuation, the films were transferred back to the dry air-filled glovebox, and a mixed organic cation solution was dynamically spin-coated at 2300 rpm for 30 s to form a wet the precursor film. The as-coated precursor film was placed onto a 70 °C hot plate to anneal for 1 min then transferred onto a 150 °C hot plate to anneal for 15 min in dry air condition.

For the ETL deposition, the perovskite films were transferred to nitrogen-filled glovebox. The PCBM solution was spin-coated at a speed of 2000 rpm for 30 s with an acceleration of 1000 rpm/s. The PCBM-coated films were kept bench dry for 10 min. After that the hot BCP solution was spin-coated at 4000 rpm for 30 s with an acceleration of 1000 rpm/s as a hole blocking layer. Finally, 80 nm Ag was deposited under a vacuum of 4 × 10^–7^ mbar. The device area was defined as 4.5 mm^2^ by a metal shadow mask.

### 5.7 Device and Film Characterization

Current density–voltage (*J*–*V*) measurements were performed in an ambient condition under simulated AM 1.5 light with an intensity of 100 mW cm^–2^ (ABET TECHNOLOGIES Sun3000 AAA solar simulator). The intensity was calibrated using a Si reference cell (NIST traceable, VLSI) and corrected by determining the spectral mismatch between the solar spectrum, reference cell, and spectral response of the device. Devices were scanned using a Keithley 2450 source meter unit from −0.5 to 1.2 V and back, with a step size of 0.05 V and a dwell time of 0.1 s. The pixel area was 3 mm × 1.5 mm. The external quantum efficiency (EQE) spectra were recorded using the monochromatic light of halogen lamp from 350 to 900 nm, the reference spectra were calibrated using the NIST-traceable Si diode (Thorlabs). Reverse-bias *J*–*V* measurements were carried out using a Keithley 2450 source meter. During the measurement, a voltage sweep ranging from +1.2 V to −11 V was applied to the ITO electrode, while the Au electrode was held at ground potential (0 V). The voltage was incremented in steps of 0.05 V, with a scan rate of 50 mV/s. Scanning electron microscopy (ZEISS Gemini 500, Oberkochen, Germany) with an acceleration voltage of 1.5 kV and the InLens, ESB, and HE-SE2 detectors were utilized to obtain the surface morphology images. Atomic force microscopy (AFM) measurement was performed with a Dimension ICON3 scanning probe microscope from Bruker AXS S.S.S under ambient conditions in the ScanAsyst mode in air using RTESPA-150 tips. The size of the recorded images was 5 by 5 μm and the scan rate was 0.5 Hz at 1024 points per line. The samples were transferred to an ultrahigh vacuum chamber (ESCALAB 250Xi by Thermo Scientific, base pressure: 1 × 10^–10^ mbar) for X-ray photoemission spectroscopy (XPS). XPS measurement was carried out using an XR6 monochromated Al kα source (*h*υ = 1486.6 eV) using a spot size of 650 μm. Pass energy of 100 and 20 eV were used for the survey and core level spectra, respectively. Ultraviolet photoelectron spectroscopy (UPS) was carried out using a double-differentially pumped He discharge lamp (*h*υ = 21.22 eV) with a pass energy of 2 eV and a bias at – 5 V. The crystal structure of the TrCa films was studied by means of X-ray diffraction (XRD). 1D XRD patterns were recorded using D8 Advance diffractometer (Bruker, USA), 2D XRD and GI-XRD patterns were recorded on SmartLab diffractometer (Rigaku, Japan) in standard θ–2θ parallel beam setup and with incident angle ω = 0.3° for surface-sensitive grazing incidence XRD.

KPFM was measured on spin-coated HTL with PPP-EFM cantilever (Nanosensors, Switzerland) with a nominal spring constant of *k* = 2.8 N/m and a conductive PtIr coating on an MFP3D (Asylum Research, USA) with an external HF2 lock-in amplifier (Zurich Instruments, Switzerland) using a heterodyne detection scheme: the electrostatic AC excitation at 1.5 V amplitude was done on the difference of the second and first eigenmode of the cantilever and the detection on the second eigenmode.^50^ Each measurement series was conducted with the same cantilevers. C-AFM measurements on spin-coated HTL films were performed in PeakForce TUNA mode on a Dimension Icon AFM (Bruker, USA). A PPM-EFM cantilever was used. During the Peak Force TUNA, the cantilever oscillates with a frequency of 1 kHz and the current is detected during the time of contact between tip and sample using a current amplifier at a sensitivity of 1 nA/V. All measurements were performed consecutively with the same C-AFM tip at a bias of 650 mV, after compensating the instrument inherent offset of −7.5 mV. Quasi-Fermi level splitting (QFLS) was determined by measuring the absolute photoluminescence quantum yield (PLQY) with an integrating sphere system (Quantiflux, Quantum Yield Berlin) linked to a calibrated spectrometer. Samples were illuminated at a one-sun equivalent. Time-resolved photoluminescence (TRPL) were performed in a PicoQuant FluoTime300 spectrometer, using a 515 nm laser as an excitation source with a repetition rate of 0.1 MHz. For the measurement, the samples were positioned at 30° in relation to the excitation laser.

## Supplementary Material



## Data Availability

The data that support the findings of this study are available from the corresponding author upon reasonable request.

## Notes

Photovoltaic parameter deviations; illuminated *J*–*V* characteristics; MPP tracking; SEM top-view morphology and cross-section images; AFM images; θ–2θ XRD patterns; 2D-GIXRD patterns; XPS; UPS; absorption spectra; photoluminescence spectra; time-resolved photoluminescence decay profile; quasi-Fermi level splitting; and KPFM surface potential (PDF)
