# Supplementary material for: Suppression of Interfacial Loss Pathways at Self-Assembled Molecular Hole Transport Layers in Perovskite Solar Cells
Source: ACS Appl Mater Interfaces. 2026 Apr 21;18(17):24604–14. doi: 10.1021/acsami.6c02006 (PMC13154134; doi:10.1021/acsami.6c02006)
Supplement: Supplementary file 1 [file am6c02006_si_001.pdf]

Supporting Information

Suppression of Interfacial Loss Pathways at Self-Assembled Molecular Hole Transport Layers in Perovskite Solar Cells

Shivam Singh,[1,2] Raquel Dantas Campos,[1,2] Marielle Deconinck,[1,2] Elena Siliavka,[1,2] Vladimir Shilovskikh,[1,2] Dmitrii Sychev,[3] Ilka M. Hermes,[3] Nir Tessler,[4] Boris Rivkin[1,2] and Yana Vaynzof [1,2]*

[1]Chair for Emerging Electronic Technologies, TUD Dresden University of Technology, Nöthnitzer Str. 61, 01187 Dresden, Germany.

[2]Leibniz-Institute for Solid State and Materials Research Dresden, Helmholtzstraße 20, 01069 Dresden, Germany.

[3] Leibniz-Institut für Polymerforschung Dresden e.V., Hohe Straße 6, Dresden D-01069, Germany

[4] Sara and Moshe Zisapel Nanoelectronic Center, Electrical and Computer Engineering, Technion Israel Institute of Technology, Haifa 32000003, Israel

Corresponding author email: y.vaynzof@ifw-dresden.de

This file will include:

Figure S1 to S16

Supplementary Note 1

Table S1 and S2

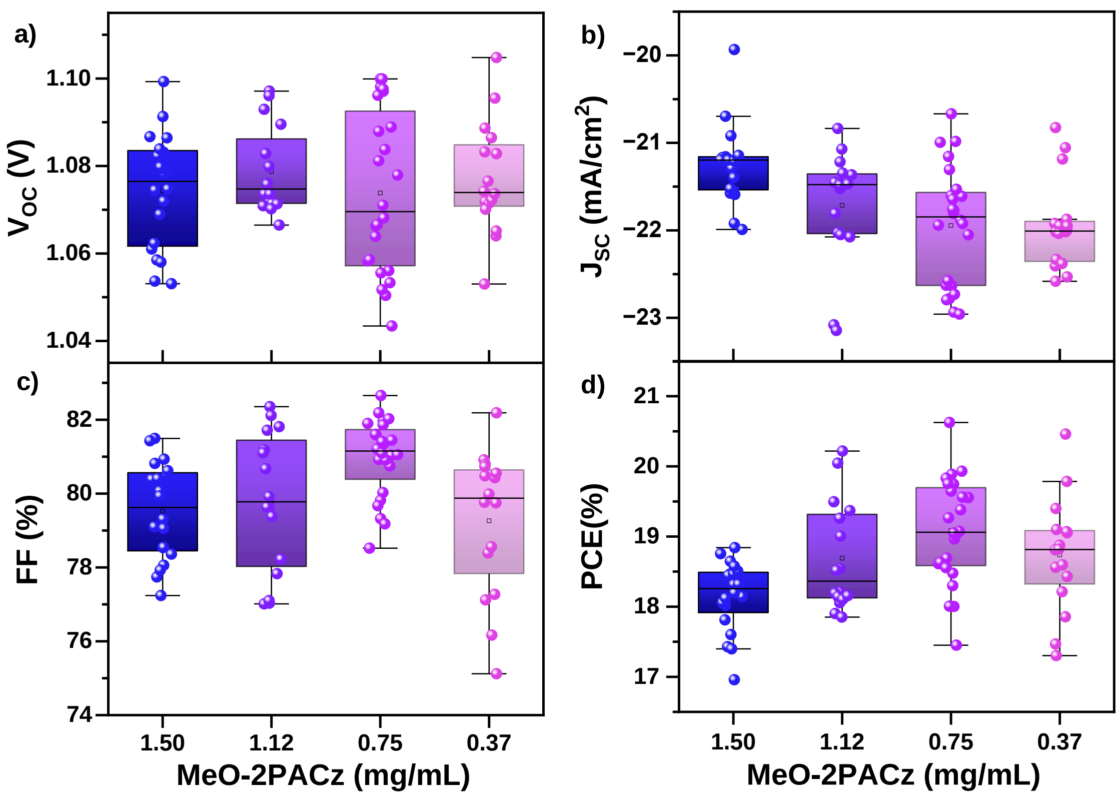

Figure S1: a) V_{OC}, b) J_{SC}, c) fill factor (FF) and d) PCE of the PSCs with MeO-2PACz as HTL with different concentration.

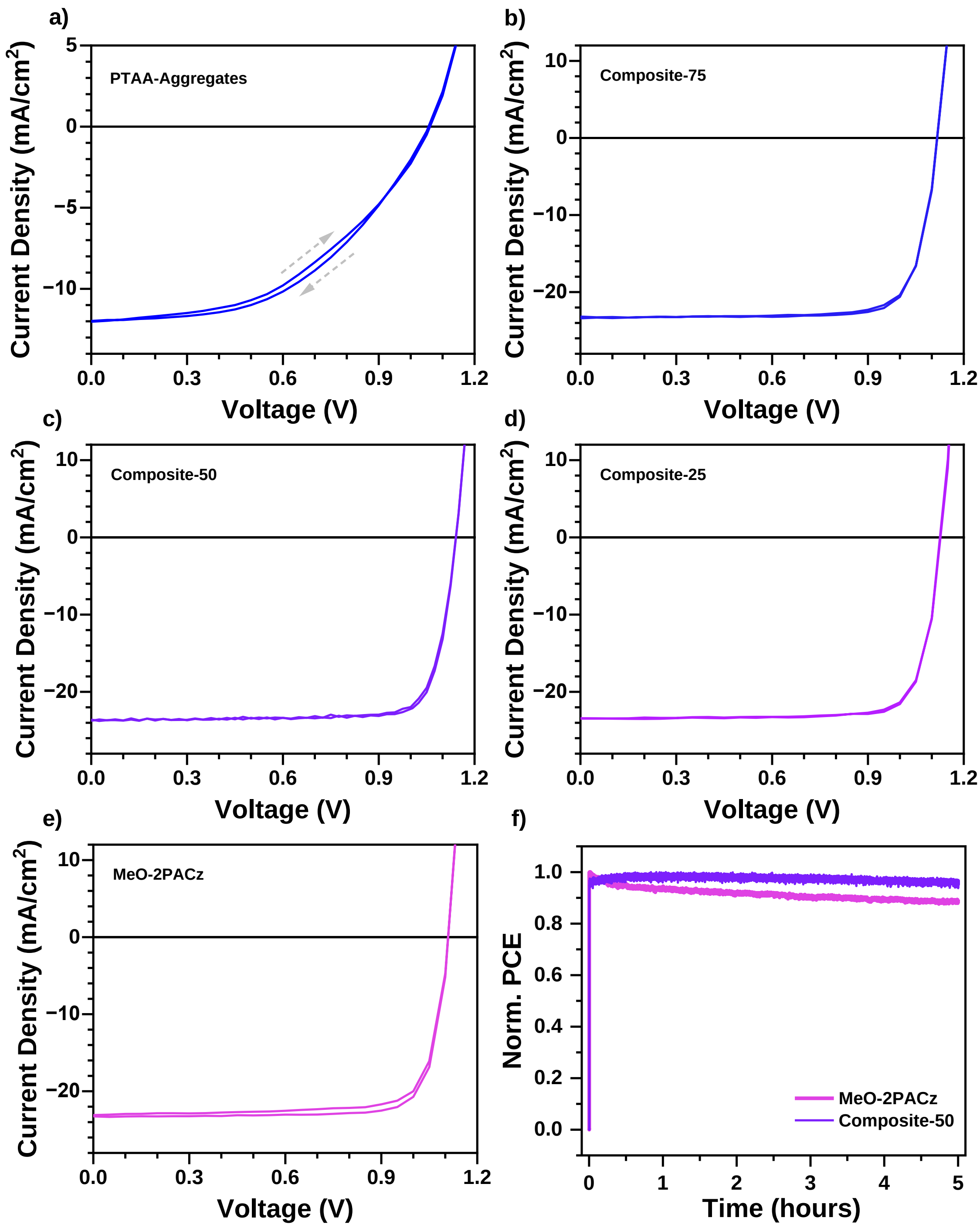

Figure S2: Illuminated J-V characteristics of PSCs with HTL as a) PTAA , b) composite-75 , c) composite-50, d) composite-25, e) MeO-2PACz. f) Maximum-power-point tracking of non-encapsulated PSC with HTL as composite-50 and MeO-2PACz.

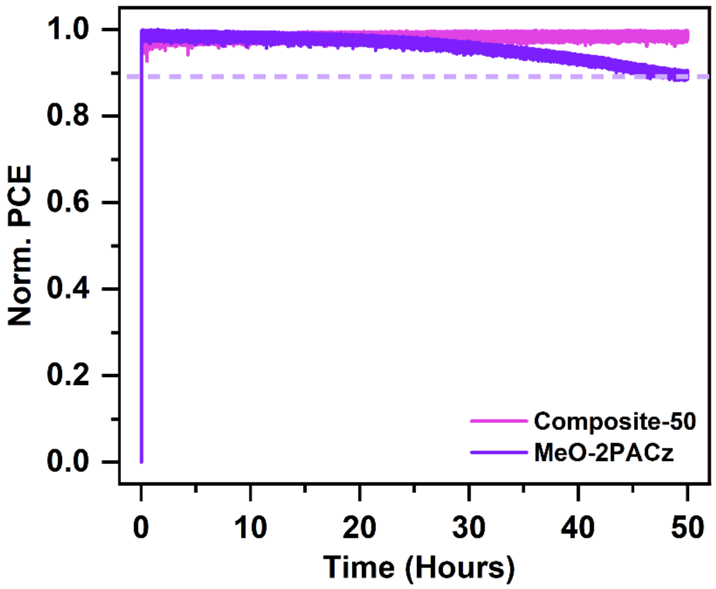

Figure S3: Maximum-power-point tracking of encapsulated PSC with HTL as composite-50 and MeO-2PACz.

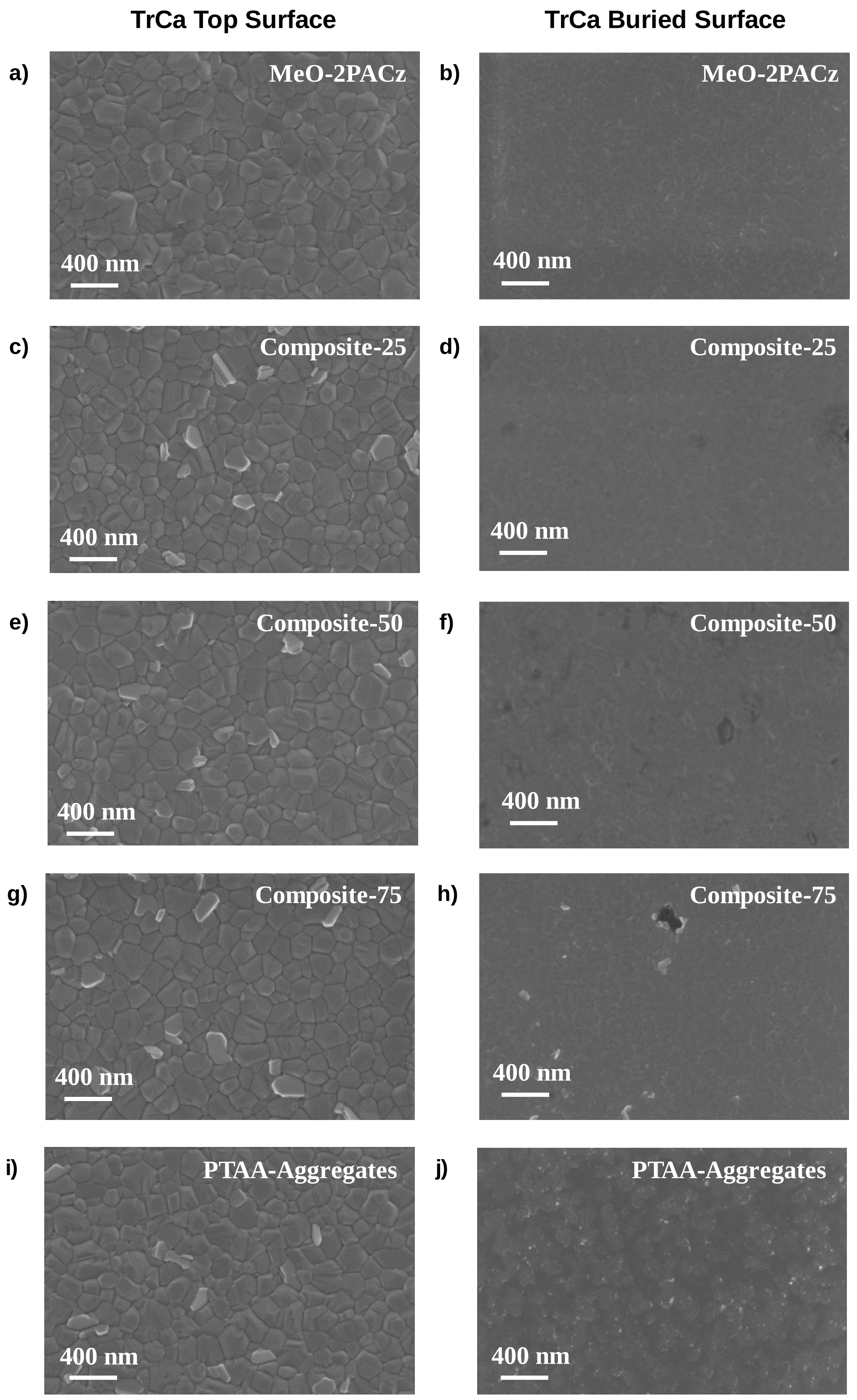

Figure S4: a,c,e,g,i) Top-view and b,d,f,h,j) buried surface morphology of TrCa perovskite absorber layer fabricated/delaminated over/from a,b) MeO-2PACz, c,d) composite-25, e,f) composite-50, g,h) composite-75 and i,j) PTAA-aggregates HTL.

The buried interface surface observed results from mechanical stress applied to the perovskite film during the delamination process from HTL-coated ITO substrates, as noted in our previous studies and in the literature.[1–3] This characterization does not necessarily indicate complete or incomplete surface coverage; instead, it shows the morphological features (texture) of the buried interface. Additionally, as the PTAA concentration in the composite HTL increases, the size of PTAA aggregates increases, likely due to PTAA's limited solubility in DMF. Therefore, the features seen at the buried interface mainly reflect the size of PTAA aggregates, rather than directly indicating the intrinsic quality of the buried interface.

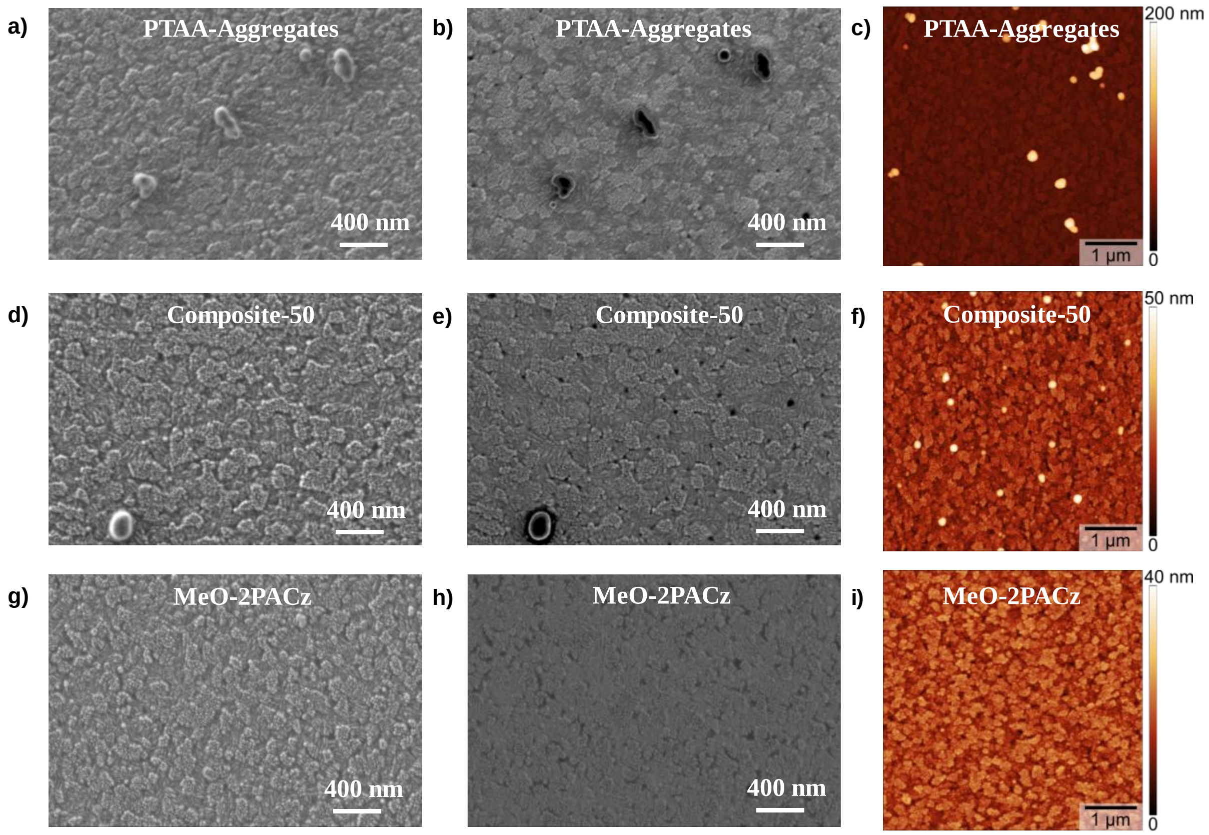

Figure S5: Top-view morphology of a,b) PTAA-aggregates, d,e) composite-50 and g,h) MeO-2PACz HTL in a,d,g) HE-SE2 and b,e,h) In-lens mode of scanning electron microscopy (SEM). AFM images of c) PTAA-aggregates, f) composite-50 and i) MeO-2PACz HTL.

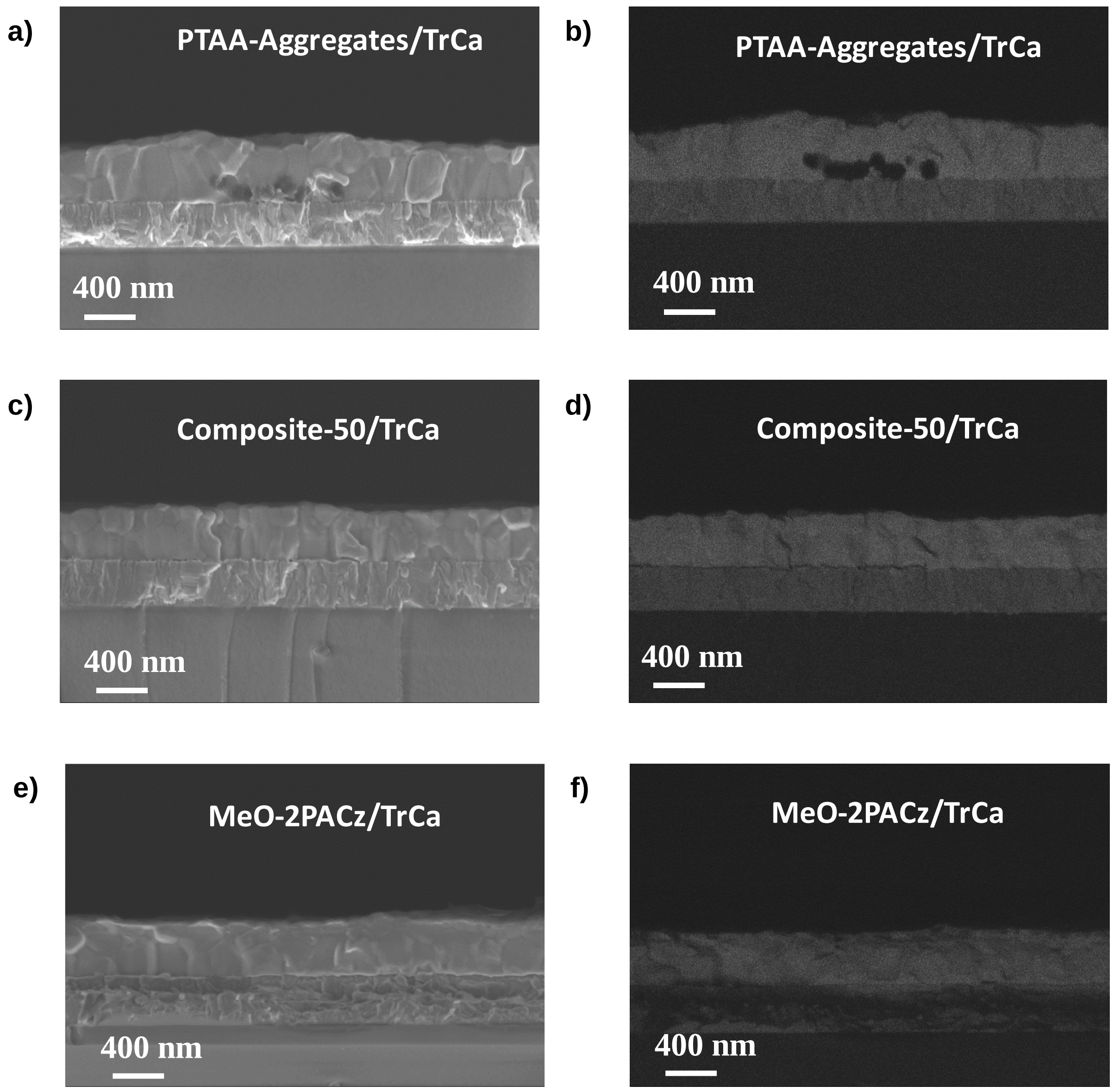

Figure S6: Cross-section SEM images of a,b) PTAA-aggregates/TrCa, c,d) composite-50/TrCa and e,f) MeO-2PACz/TrCa in a,c,e) In-lens and b,d,f) ESB mode of scanning electron microscopy (SEM).

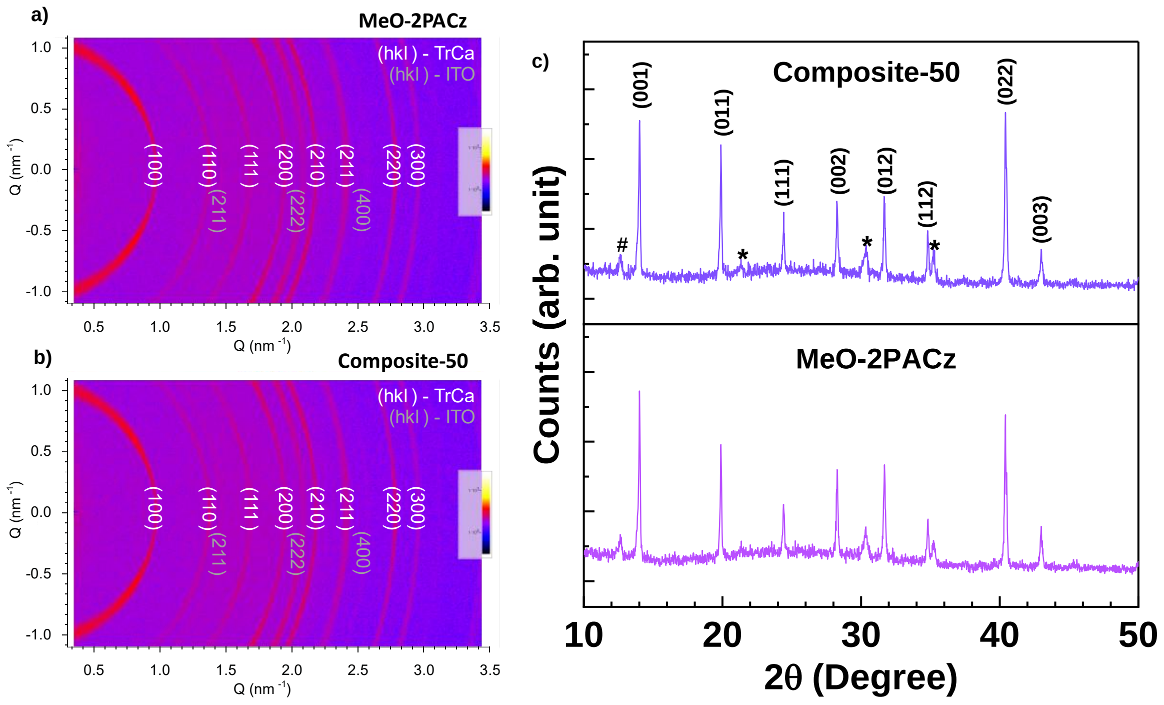

Figure S7: θ-2θ XRD patterns of TrCa perovskite film fabricated on a,c) MeO-2PACz and b,d) composite-50 HTL. Here, # = PbI_2 and * = ITO.

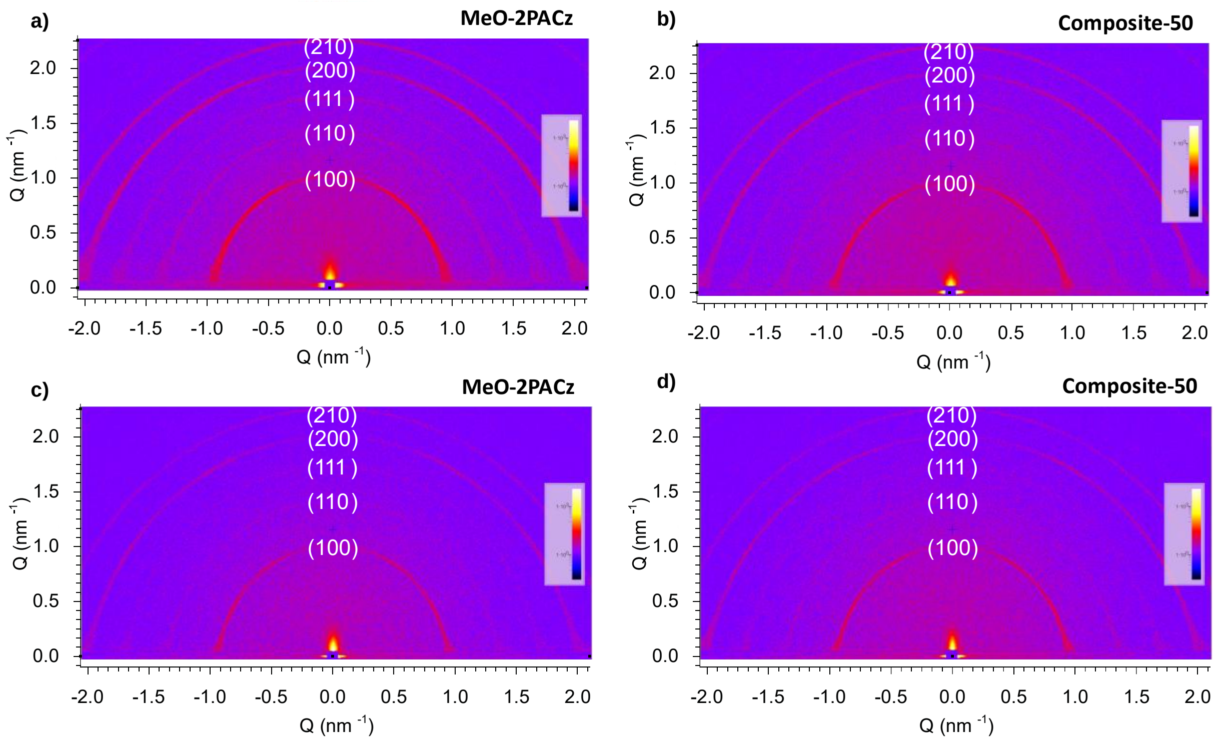

Figure S8: 2D-GIXRD patterns, at X-ray incident angles of 0.3° with surface sensitivity, of the a,b) top and c,d) bottom surfaces of TrCa perovskite films fabricated on a,c) MeO-2PACZ and b,d) composite-50 HTL.

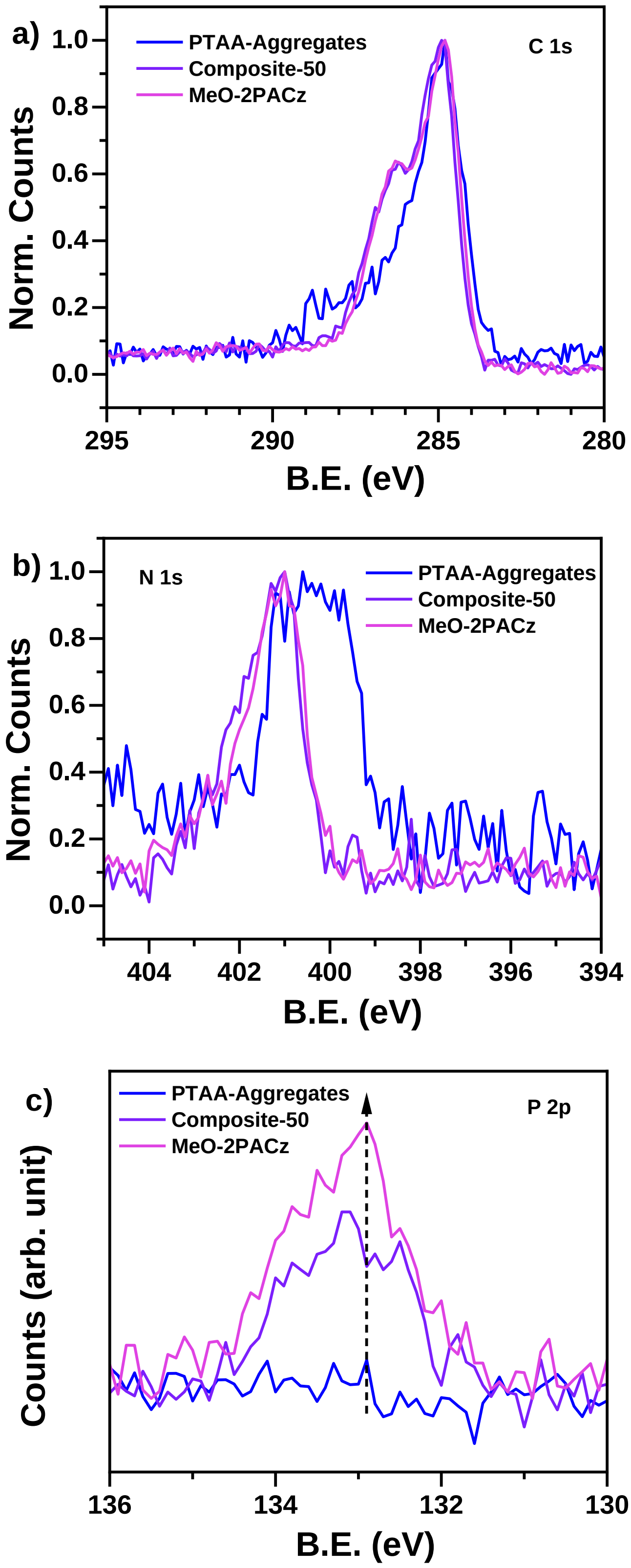

Figure S9: The core-level spectra of a) C 1s, b) N 1s, and c) P 2p measured on PTAA-aggregates, composite-50 and MeO-2PACz HTLs.

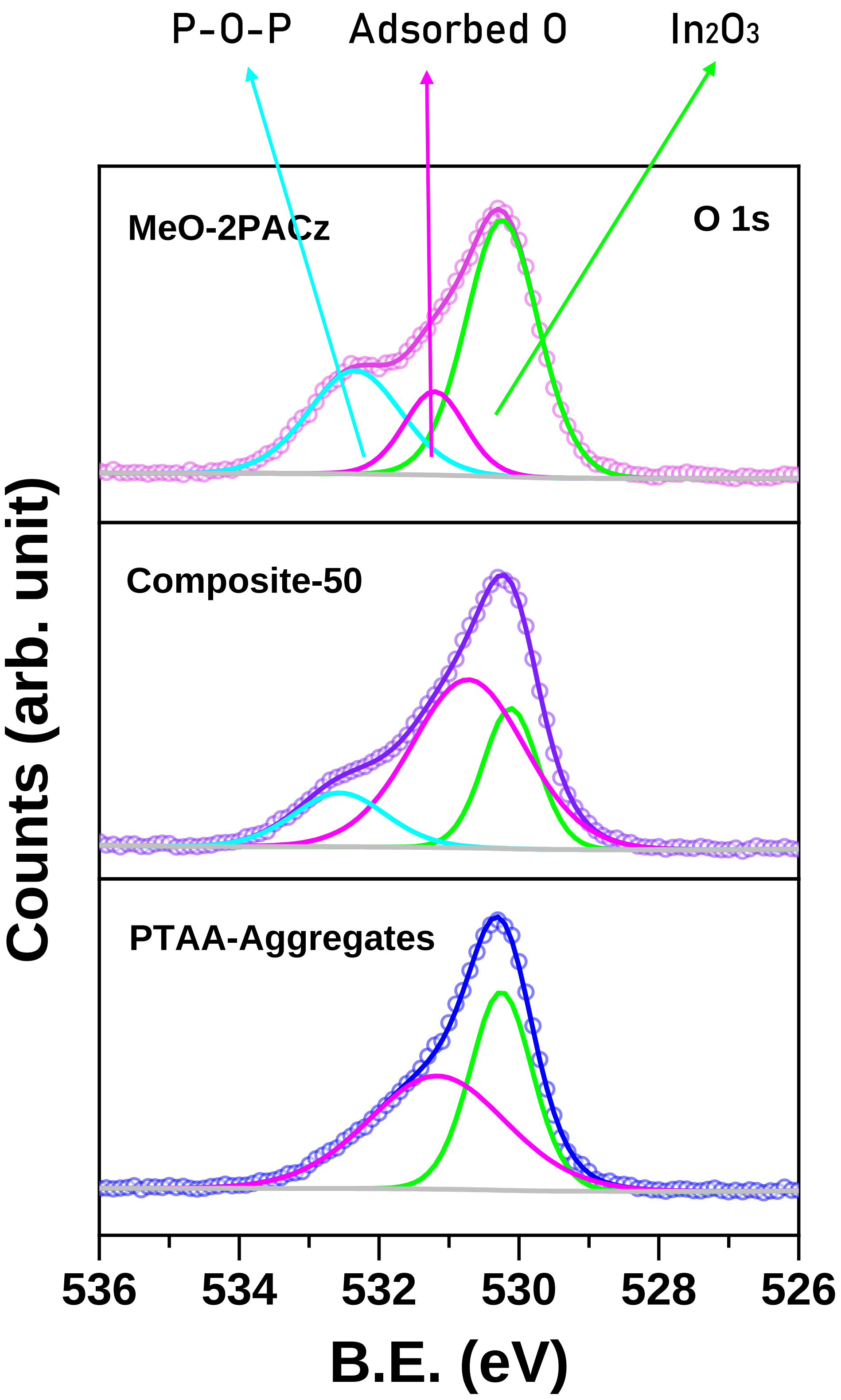

Figure S10: The core-level spectra of O 1s measured on PTAA-aggregates, composite-50 and MeO-2PACz HTLs.

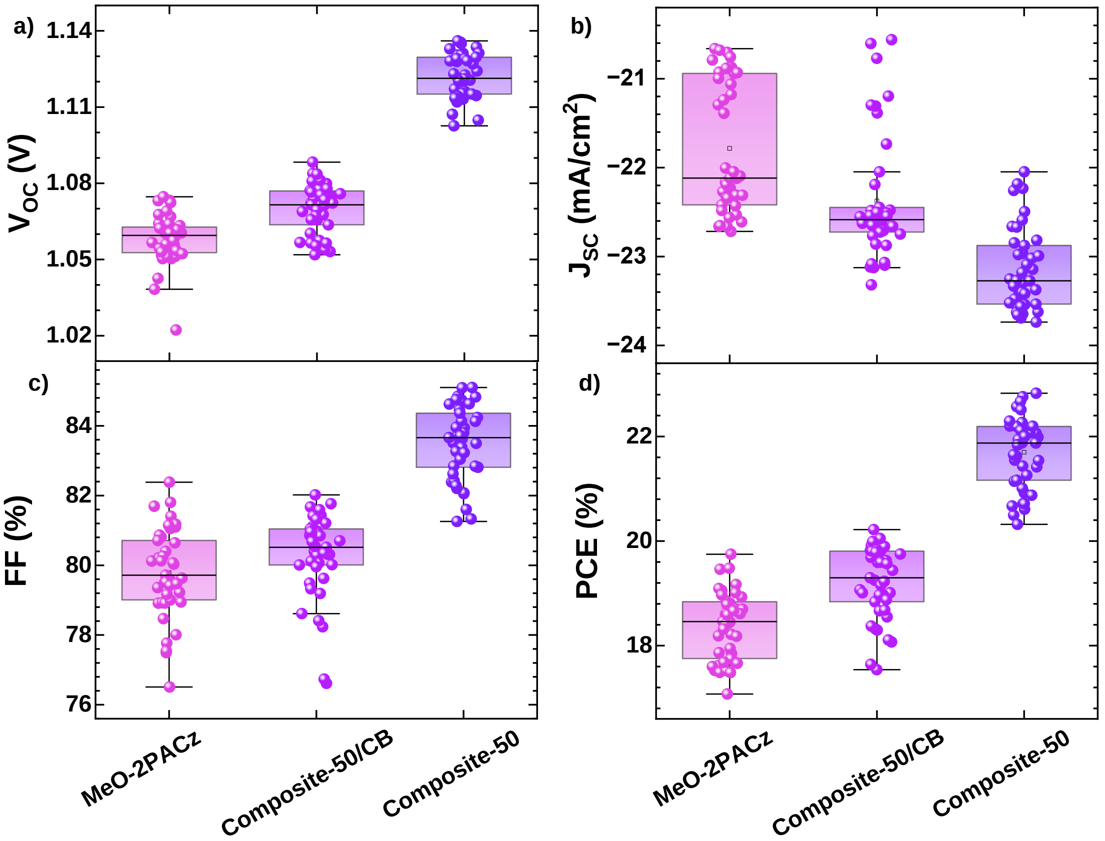

Figure S11: a) V_{OC}, b) J_{SC}, c) FF and d) PCE of the PSCs with HTL as MeO-2PACz, composite-50 and composite-50 washed by chlorobenzene.

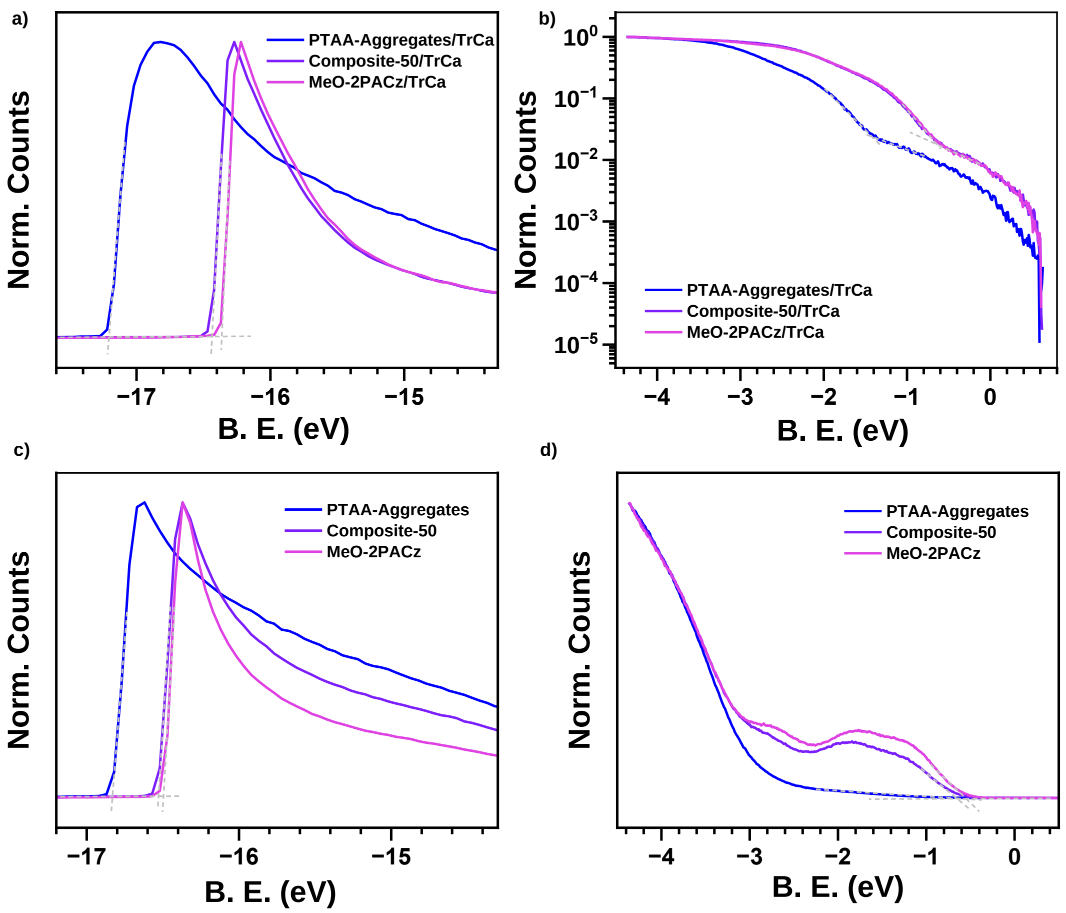

Figure S12: UPS spectra of a,c) the photoemission onset and b,d) valence band of a,b) TrCa perovskite films and c,d) HTLs.

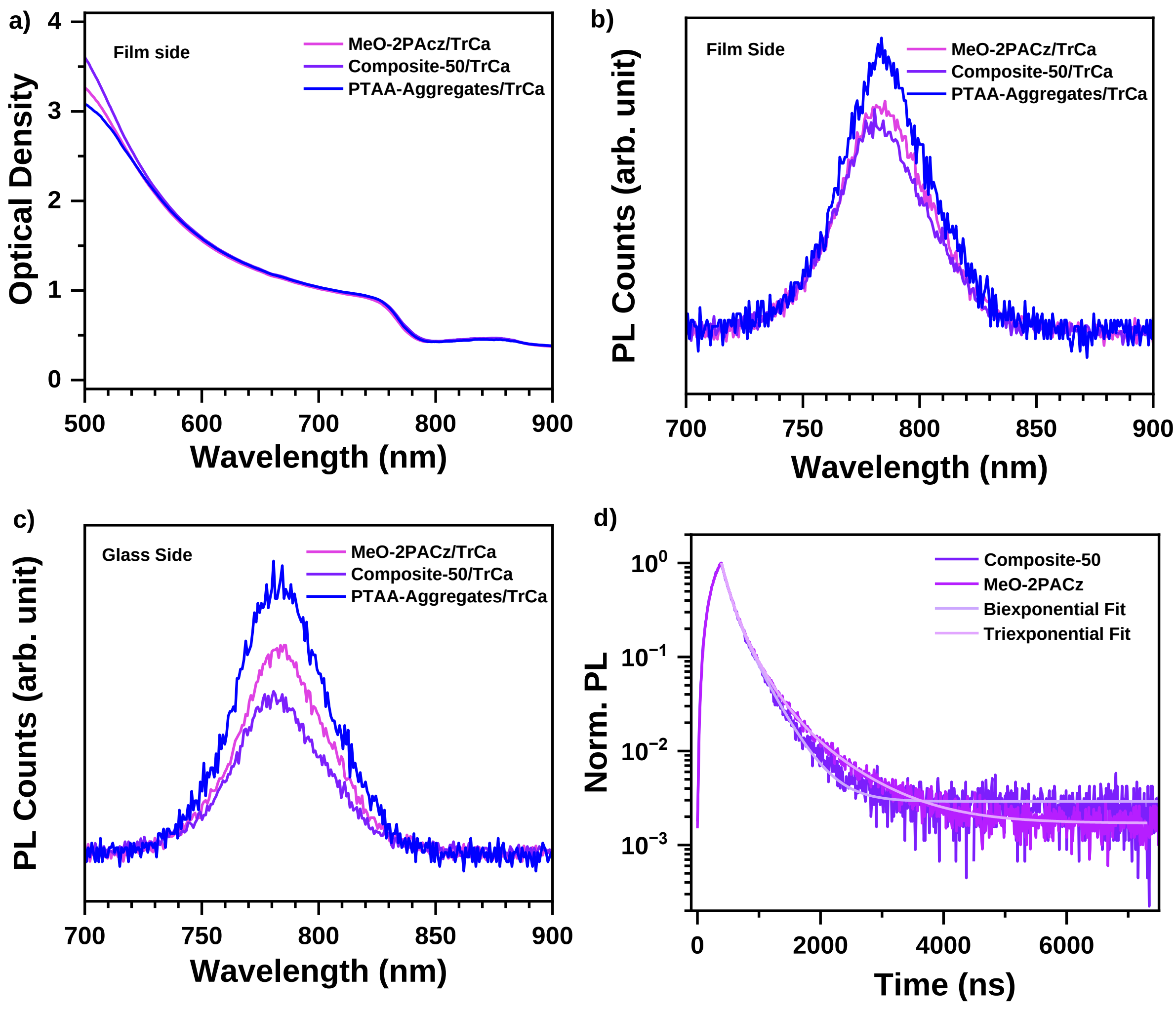

Figure S13: a) Absorption and b,c) photoluminescence (PL) of TrCa perovskite film fabricated over the HTLs: PTAA-aggregates, composite-50 and MeO-2PACz. d) Time-resolved photoluminescence (TROL) decay profile of TrCa perovskite film fabricated over the composite-50 and MeO-2PACz HTLs.

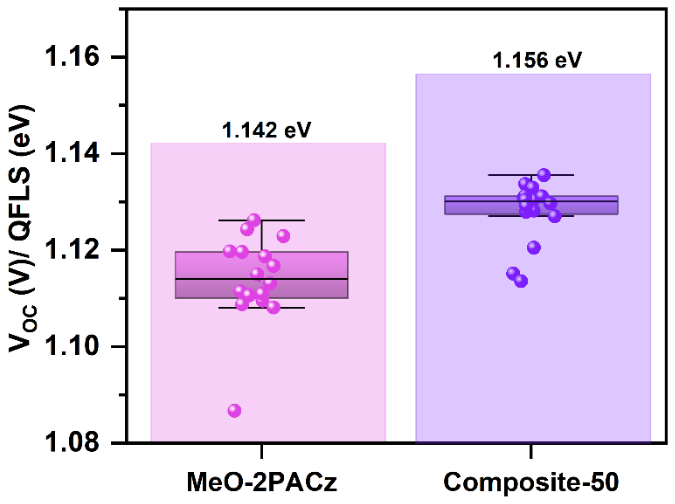

Figure S14: Comparison of the QFLS values (the columns) for half-completed devices (up to the TrCa perovskite layer) with the measured Voc of completed devices (the spheres).

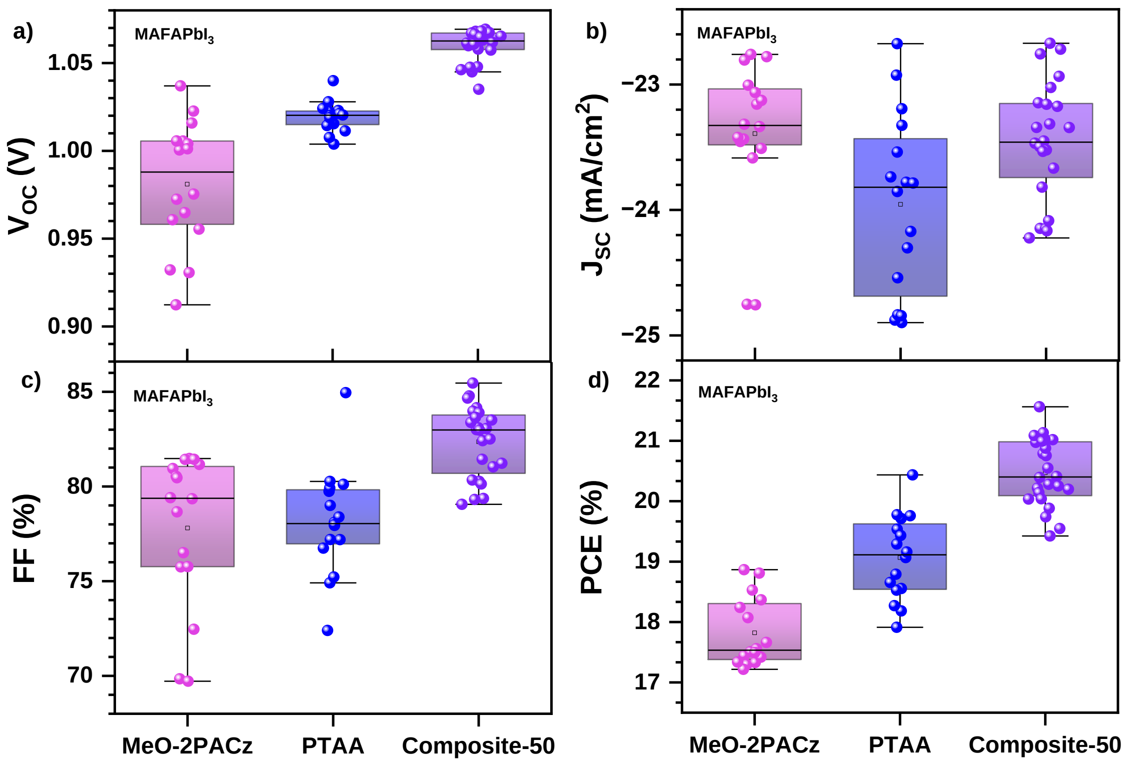

Figure S15: a) V_{OC}, b) J_{SC}, c) FF and d) PCE of the two-step deposited $MAFAPbI_3$ based-PSCs with HTL as MeO-2PACz, PTAA and composite-50. Here, PTAA is dissolved in chlorobenzene with a 1.5 mg/mL concentration.

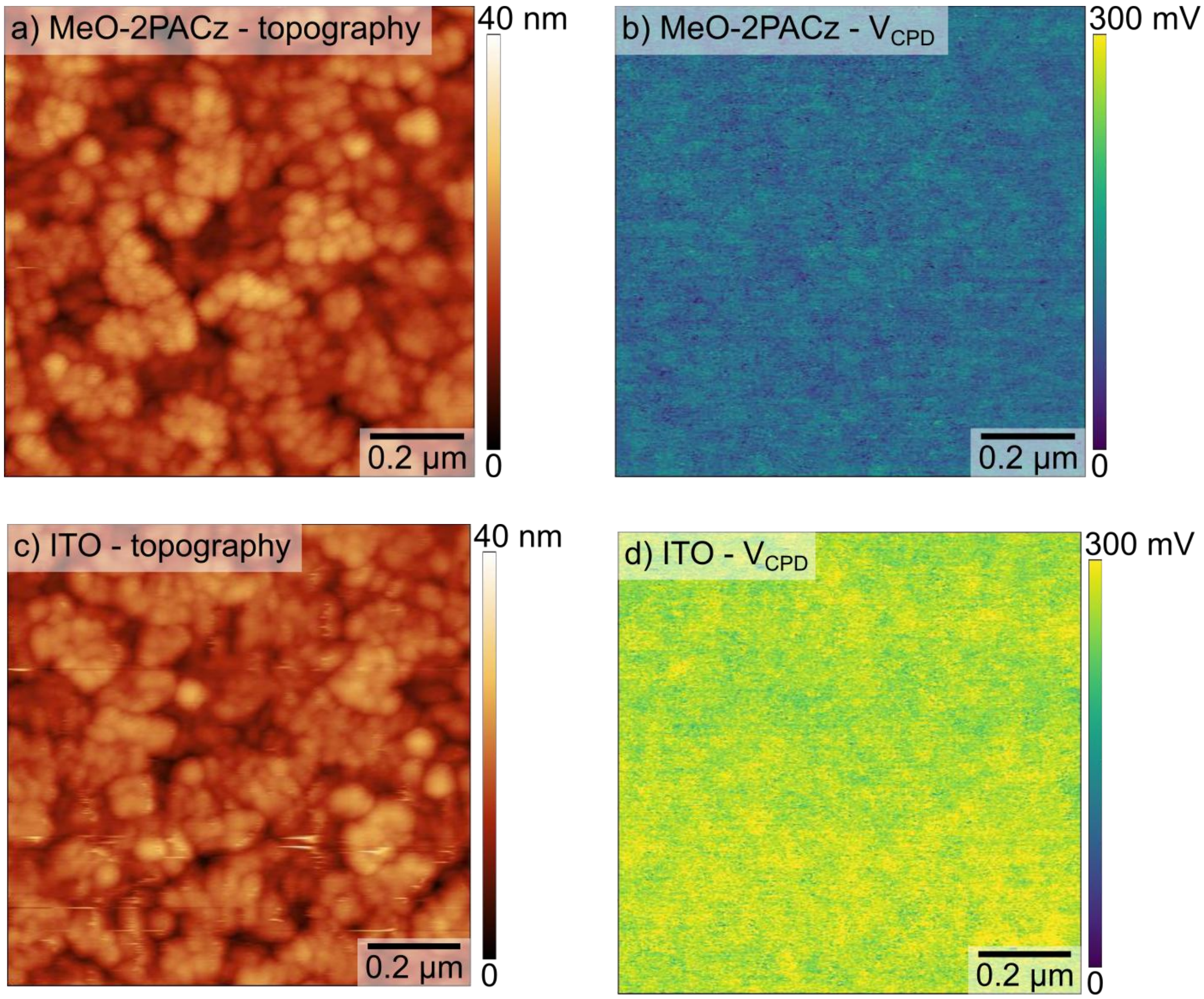

Figure S16: Enlarged image details of a, c) AFM topography and b, d) KPFM surface potential (V_{CPD}) of a, b) MeO-2PACz, and c, d) bare ITO as reference. The average V_{CPD} for MeO-2PACz and ITO is 110 mV and 270 mV, respectively.

Supplementary Note 1

To simulate the J-V under one Sun, we have used the same simulation tool described in the references.[4–6] In short, we utilize the Sentaurus device by Synopsys, which solves the drift-diffusion and Poisson equations self-consistently for electrons, holes, and ions. The ions exist and are mobile only within the perovskite layer, while the electrons and holes are free to move within the transporting/blocking layers. The ions are implemented using the "hydrogen diffusion" module, and tunneling across the thin HTL was introduced using "nonlocal tunneling". To produce the J-V curves, we solve at each bias point for the steady state of both ions and electronic charges. This would mimic a slow scan that cancels any small hysteresis, allowing for focusing on the curves' shape.

The simulation parameters are listed below, where, for simplicity, we assumed the organic layers to be relatively ordered (σ = 3kT) and the effective mass for tunneling was taken to be on the high side (0.4). The TCO is modeled as an electrode. The tunneling is to and from this electrode.

	**Perovskite**	**PCBM**	**HTL**
μ_e $cm^2V^{-1}s^{-1}$	5 [7,8]	10^{-3}	10^{-5}
μ_h $cm^2V^{-1}s^{-1}$	5 [7]	10^{-5}	10^{-3}
LUMO eV	4.0	4.0	2.1
HOMO eV	5.6	6.0	5.3
LUMO Effective DOS cm^{-3}	$7x10^{18}$ [9]	10^{21}	10^{21}
HOMO Effective DOS cm^{-3}	$2.5x10^{18}$ [9]	10^{21}	10^{21}
σ meV	--	78	78
Bimolecular coef cm^3s^{-1}	10^{-10} [10]	Langevin	Langevin
epsilon	30 [11]	3	3
Length nm	450	40	variable
Mobile ions	Negative only		
Ions density cm^{-3}	$1x10^{18}$ [5]		
Electron effective mass for tunnelling			0.4 [12–14]

Table S1: Photovoltaic performance parameters from recent publications of inverted perovskite solar cells utilizing either PTAA or MeO-2PACz as a single HTL, combined with triple-cation mixed-halide perovskite absorbers, without any additional surface passivation or interfacial modification.

**HTL**	**V_{OC} (V)**	**J_{SC} (mA/cm²)**	**FF (%)**	**PCE (%)**	**References**
PTAA	1.09	21.26	78.30	18.14	[15]
PTAA	1.10	21.99	76.36	18.50	[16]
PTAA	1.13	22.06	73.78	18.36	[17]
PTAA	1.06	20.75	75.10	17.21	[18]
PTAA	1.10	22.87	81.35	20.47	[1]
PTAA	1.10	23.33	83.40	21.58	[19]
PTAA	1.08	22.74	78.00	19.17	[20]
MeO-2PACz	1.12	22.25	83.64	20.93	[1]
MeO-2PACz	1.07	23.20	79.00	19.60	[21]
MeO-2PACz	1.10	23.60	82.12	21.38	[22]
MeO-2PACz	1.12	21.70	77.50	19.00	[23]
MeO-2PACz	1.14	22.20	80.50	20.40	[24]
MeO-2PACz	1.12	21.70	77.50	20.42	[25]
MeO-2PACz	1.09	23.52	79.36	20.35	[26]
MeO-2PACz	1.14	22.76	78.64	20.40	[27]
MeO-2PACz	1.12	23.02	78.00	20.11	[28]
MeO-2PACz	1.12	22.69	81.87	20.81	This work
Composite-50	1.14	23.64	84.68	**22.83**	**This work**

Table S2: Summary of time-resolved photoluminescence (TRPL) fitting parameters for perovskite films deposited on MeO-2PACz and composite-50 HTLs.

HTLs	A_1	τ_1 (ns)	A_2	τ_2 (ns)	A_3	τ_3 (ns)	$\tau_{av.}$ (ns)
**MeO-2PACz**	0.10	288.9	0.89	113.2	0.01	756.1	137
**Composite-50**	0.92	124.3	0.08	359.7	-	-	144

References:

(1) Singh, S.; Siliavka, E.; Löffler, M.; Vaynzof, Y. Impact of Buried Interface Texture on Compositional Stratification and Ion Migration in Perovskite Solar Cells. *Adv. Funct. Mater.* **2024**, *34* (42), 2402655. https://doi.org/10.1002/ADFM.202402655.

(2) Dai, Z.; Yadavalli, S. K.; Chen, M.; Abbaspourtamijani, A.; Qi, Y.; Padture, N. P. Interfacial Toughening with Self-Assembled Monolayers Enhances Perovskite Solar Cell Reliability. *Science (80-.).* **2021**, *372* (6542), 618–622. https://doi.org/10.1126/science.abf5602.

(3) Chen, S.; Dai, X.; Xu, S.; Jiao, H.; Zhao, L.; Huang, J. Stabilizing Perovskite-Substrate Interfaces for High-Performance Perovskite Modules. *Science (80-.).* **2021**, *373* (6557), 902–907. https://doi.org/10.1126/science.abi6323.

(4) Bitton, S.; Tessler, N. Electronic-Ionic Coupling in Perovskite Based Solar Cells: Implications for Device Stability. *Appl. Phys. Lett.* **2020**, *117* (13), 133904. https://doi.org/10.1063/5.0023902/567138.

(5) Bitton, S.; Tessler, N. Perovskite Ionics – Elucidating Degradation Mechanisms in Perovskite Solar Cells via Device Modelling and Iodine Chemistry. *Energy Environ. Sci.* **2023**, *16* (6), 2621–2628. https://doi.org/10.1039/D3EE00881A.

(6) Schramm, T.; Deconinck, M.; Ji, R.; Siliavka, E.; Hofstetter, Y. J.; Löffler, M.; Shilovskikh, V. V; Brunner, J.; Li, Y.; Bitton, S.; Tessler, N.; Vaynzof, Y.; Schramm, T.; Deconinck, M.; Ji, R.; Siliavka, E.; Hofstetter, Y. J.; Shilovskikh, V. V; Brunner, J.; Li, Y.; Vaynzof, Y.; Löffler, M.; Bitton, S.; Tessler Sara, N.; Zisapel, M. Electrical Doping of Metal Halide Perovskites by Co-Evaporation and Application in PN Junctions. *Adv. Mater.* **2024**, *36* (29), 2314289. https://doi.org/10.1002/ADMA.202314289.

(7) Herz, L. M. Charge-Carrier Mobilities in Metal Halide Perovskites: Fundamental Mechanisms and Limits. *ACS Energy Lett.* **2017**, *2* (7), 1539–1548. https://doi.org/10.1021/ACSENERGYLETT.7B00276.

(8) Wehrenfennig, C.; Eperon, G. E.; Johnston, M. B.; Snaith, H. J.; Herz, L. M. High Charge Carrier Mobilities and Lifetimes in Organolead Trihalide Perovskites. *Adv. Mater.* **2014**, *26* (10), 1584–1589. https://doi.org/10.1002/ADMA.201305172;JOURNAL:JOURNAL:15214095;PAGEGROUP:STRING:PUBLICATION.

(9) Zhou, Y.; Long, G. Low Density of Conduction and Valence Band States Contribute to the High Open-Circuit Voltage in Perovskite Solar Cells. *J. Phys. Chem. C* **2017**, *121* (3), 1455–1462. https://doi.org/10.1021/ACS.JPCC.6B10914.

(10) Johnston, M. B.; Herz, L. M. Hybrid Perovskites for Photovoltaics: Charge-Carrier Recombination, Diffusion, and Radiative Efficiencies. *Acc. Chem. Res.* **2015**, *49* (1), 146–154.

https://doi.org/10.1021/ACS.ACCOUNTS.5B00411.

(11) Anusca, I.; Balčiūnas, S.; Gemeiner, P.; Svirskas, Š.; Sanlialp, M.; Lackner, G.; Fettkenhauer, C.; Belovickis, J.; Samulionis, V.; Ivanov, M.; Dkhil, B.; Banys, J.; Shvartsman, V. V.; Lupascu, D. C. Dielectric Response: Answer to Many Questions in the Methylammonium Lead Halide Solar Cell Absorbers. *Adv. Energy Mater.* **2017**, *7* (19), 1700600. https://doi.org/10.1002/AENM.201700600;ISSUE:ISSUE:DOI.

(12) Guo, Z.; Sato, T.; Han, Y.; Takamura, N.; Ikeda, R.; Miyamoto, T.; Kida, N.; Ogino, M.; Takahashi, Y.; Kasuya, N.; Watanabe, S.; Takeya, J.; Wei, Q.; Mukaida, M.; Okamoto, H. Band Transport Evidence in PEDOT:PSS Films Using Broadband Optical Spectroscopy from Terahertz to Ultraviolet Region. *Commun. Mater.* **2024**, *5* (26), 1-13. https://doi.org/10.1038/s43246-024-00451-1.

(13) Arkhipov, V. I.; Heremans, P.; Bässler, H. Why Is Exciton Dissociation so Efficient at the Interface between a Conjugated Polymer and an Electron Acceptor? *Appl. Phys. Lett.* **2003**, *82* (25), 4605–4607. https://doi.org/10.1063/1.1586456.

(14) Che, H. J.; Chia, P. J.; Chua, L. L.; Sivaramakrishnan, S.; Tang, J. C.; Wee, A. T. S.; Chan, H. S. O.; Ho, P. K. H. Robust Reproducible Large-Area Molecular Rectifier Junctions. *Appl. Phys. Lett.* **2008**, *92* (25), 253503. https://doi.org/10.1063/1.2940592/335050.

(15) Tan, Y.; Chang, X.; Zhong, J. X.; Feng, W.; Yang, M.; Tian, T.; Gong, L.; Wu, W. Q. Chemical Linkage and Passivation at Buried Interface for Thermally Stable Inverted Perovskite Solar Cells with Efficiency over 22%. *CCS Chem.* **2023**, *5* (8), 1802–1814. https://doi.org/10.31635/CCSCHEM.022.202202154/SUPPL_FILE/CCSC-2022-02154-FILE002.DOCX.

(16) Murugan, S.; Liu, X.; Lee, E. C. Improved Current Density of Inverted Perovskite Solar Cells via Hole Transport Layer Doping. *J. Mater. Chem. C* **2024**, *12* (20), 7278–7285. https://doi.org/10.1039/D4TC00053F.

(17) Li, Y.; Wang, B.; Liu, T.; Zeng, Q.; Cao, D.; Pan, H.; Xing, G. Interfacial Engineering of PTAA/Perovskites for Improved Crystallinity and Hole Extraction in Inverted Perovskite Solar Cells. *ACS Appl. Mater. Interfaces* **2022**, *14* (2), 3284–3292. https://doi.org/10.1021/ACSAMI.1C21000/ASSET/IMAGES/LARGE/AM1C21000_0005.JPEG.

(18) Hu, L.; Zhang, L.; Ren, W.; Zhang, C.; Wu, Y.; Liu, Y.; Sun, Q.; Dai, Z.; Cui, Y.; Cai, L.; Zhu, F.; Hao, Y. High Efficiency Perovskite Solar Cells with PTAA Hole Transport Layer Enabled by PMMA:F4-TCNQ Buried Interface Layer. *J. Mater. Chem. C* **2022**, *10* (26), 9714–9722. https://doi.org/10.1039/D2TC01494G.

(19) Degani, M.; An, Q.; Albaladejo-Siguan, M.; Hofstetter, Y. J.; Cho, C.; Paulus, F.; Grancini, G.; Vaynzof, Y. 23.7% Efficient Inverted Perovskite Solar Cells by Dual Interfacial Modification. *Sci. Adv.* **2021**, *7* (49), 7930. https://doi.org/10.1126/SCIADV.ABJ7930/SUPPL_FILE/SCIADV.ABJ7930_SM.PDF.

(20) Bagheri, Z.; Matteocci, F.; Lamanna, E.; Di Girolamo, D.; Marrani, A. G.; Zanoni, R.; Di Carlo, A.; Moshaii, A. Light-Induced Improvement of Dopant-Free PTAA on Performance of Inverted Perovskite Solar Cells. *Sol. Energy Mater. Sol. Cells* **2020**, *215*, 110606. https://doi.org/10.1016/J.SOLMAT.2020.110606.

(21) Guo, Y.; Huang, L.; Wang, C.; Huang, J.; Liu, S.; Liu, X.; Zhang, J.; Hu, Z.; Zhu, Y. Efficient Inverted Perovskite Solar Cells with a Low-Temperature Processed NiOx/SAM Hole Transport Layer. *J. Mater. Chem. C* **2024**, *12* (4), 1507–1515. https://doi.org/10.1039/D3TC03575A.

(22) Jiang, T.; Yang, Y.; Hao, X.; Fan, J.; Wu, L.; Wang, W.; Zeng, G.; Halim, M. A.; Zhang, J. Self-Assembled Monolayer Hole Transport Layers for High-Performance and Stable Inverted Perovskite Solar Cells. *Energy and Fuels* **2024**, *38* (21), 21371–21381.

https://doi.org/10.1021/ACS.ENERGYFUELS.4C03889/ASSET/IMAGES/LARGE/EF4C03889_0005.JPEG.

(23) Phung, N.; Verheijen, M.; Todinova, A.; Datta, K.; Verhage, M.; Al-Ashouri, A.; Köbler, H.; Li, X.; Abate, A.; Albrecht, S.; Creatore, M. Enhanced Self-Assembled Monolayer Surface Coverage by ALD NiO in p-i-n Perovskite Solar Cells. *ACS Appl. Mater. Interfaces* **2022**, *14* (1), 2166–2176. https://doi.org/10.1021/ACSAMI.1C15860/ASSET/IMAGES/LARGE/AM1C15860_0005.JPEG.

(24) Al-Ashouri, A.; Magomedov, A.; Roß, M.; Jošt, M.; Talaikis, M.; Chistiakova, G.; Bertram, T.; Márquez, J. A.; Köhnen, E.; Kasparavičius, E.; Levcenco, S.; Gil-Escrig, L.; Hages, C. J.; Schlatmann, R.; Rech, B.; Malinauskas, T.; Unold, T.; Kaufmann, C. A.; Korte, L.; Niaura, G.; Getautis, V.; Albrecht, S. Conformal Monolayer Contacts with Lossless Interfaces for Perovskite Single Junction and Monolithic Tandem Solar Cells. *Energy Environ. Sci.* **2019**, *12* (11), 3356–3369. https://doi.org/10.1039/C9EE02268F.

(25) Zhou, G.; Hashemi, F.; Ding, C.; Luo, X.; Zhang, L.; Sheibani, E.; Luo, Q.; Jumabekov, A. N.; Österbacka, R.; Xu, B.; Ma, C. Perovskite Solar Cells Modified with Conjugated Self-Assembled Monolayers at Buried Interfaces. *Nanomaterials* **2025**, *15* (13), 1014. https://doi.org/10.3390/NANO15131014/S1.

(26) Telschow, O.; Albaladejo-Siguan, M.; Merten, L.; Taylor, A. D.; Goetz, K. P.; Schramm, T.; Konovalov, O. V.; Jankowski, M.; Hinderhofer, A.; Paulus, F.; Schreiber, F.; Vaynzof, Y. Preserving the Stoichiometry of Triple-Cation Perovskites by Carrier-Gas-Free Antisolvent Spraying. *J. Mater. Chem. A* **2022**, *10* (37), 19743–19749. https://doi.org/10.1039/D1TA10566C.

(27) Telschow, O.; Scheffczyk, N.; Hinderhofer, A.; Merten, L.; Kneschaurek, E.; Bertram, F.; Zhou, Q.; Löffler, M.; Schreiber, F.; Paulus, F.; Vaynzof, Y. Elucidating Structure Formation in Highly Oriented Triple Cation Perovskite Films. *Adv. Sci.* **2023**, *10* (17), 2206325. https://doi.org/10.1002/ADVS.202206325;WEBSITE:WEBSITE:ADVANCED;CTYPE:STRING:JOURNAL.

(28) Hossain, K.; Singh, S.; Kabra, D. Role of Monovalent Cations in the Dielectric Relaxation Processes in Hybrid Metal Halide Perovskite Solar Cells. *ACS Appl. Energy Mater.* **2022**, *5* (3), 3689–3697. https://doi.org/10.1021/ACSAEM.2C00108/ASSET/IMAGES/LARGE/AE2C00108_0005.JPEG.
